# Assessing Performance of Contemporary Plant-Based Diets against the UK Dietary Guidelines: Findings from the Feeding the Future (FEED) Study

**DOI:** 10.3390/nu16091336

**Published:** 2024-04-29

**Authors:** Izabella Lawson, Caroline Wood, Nandana Syam, Holly Rippin, Selina Dagless, Kremlin Wickramasinghe, Birdem Amoutzopoulos, Toni Steer, Timothy J. Key, Keren Papier

**Affiliations:** 1 Cancer Epidemiology Unit, Nuffield Department of Population Health, University of Oxford, Oxford OX3 7LF, UK; izabella.lawson@ndph.ox.ac.uk (I.L.); tim.key@ndph.ox.ac.uk (T.J.K.); 2 Public Affairs & Communications Directorate, University of Oxford, Oxford OX1 2JD, UK; caroline.wood@admin.ox.ac.uk; 3 Medical Sciences Division, University of Oxford, Oxford OX3 9DU, UK; nandana.syam@nhs.net; 4 Special Initiative on NCDs and Innovation, World Health Organization Regional Office for Europe, DK-2100 Copenhagen, Denmark; rippinh@who.int (H.R.); daglesss@who.int (S.D.); wickramasinghek@who.int (K.W.); 5 MRC Epidemiology Unit, University of Cambridge School of Clinical Medicine, University of Cambridge, Cambridge CB2 0SL, UK; birdem.amoutzopoulos@mrc-epid.cam.ac.uk (B.A.); toni.steer@mrc-epid.cam.ac.uk (T.S.)

**Keywords:** vegetarians, vegans, flexitarians, plant-based, diet, cohort, dietary guidelines, nutrients

## Abstract

Uncertainty remains about the composition of contemporary plant-based diets and whether they provide recommended nutrient intakes. We established Feeding the Future (FEED), an up-to-date online cohort of UK adults following different plant-based diets and diets containing meat and fish. We recruited 6342 participants aged 18–99 [omnivores (1562), flexitarians (1349), pescatarians (568), vegetarians (1292), and vegans (1571)] between February 2022 and December 2023, and measured diet using a food frequency questionnaire and free text. We compared personal characteristics and dietary intakes between diet groups and assessed compliance with dietary guidelines. Most participants met UK dietary recommendations for fruit and vegetables, sodium, and protein, although protein intakes were lowest among vegetarians and vegans. Omnivores did not meet the fibre recommendation and only vegans met the saturated fat recommendation. All diet groups exceeded the free sugars recommendation. Higher proportions of vegetarians and vegans were below the estimated average requirements (EARs) for zinc, iodine, selenium, and, in vegans, vitamins A and B12, whereas calcium intakes were similar across the diet groups. People following plant-based diets showed good compliance with most dietary targets, and their risk for inadequate intakes of certain nutrients might be mitigated by improved dietary choices and/or food fortification.

## 1. Introduction

Plant-based diets, which emphasise foods from plant sources and minimal or no intake of animal-based products, have become increasingly popular in the UK; between 2008/2009 and 2018/2019, the proportion of adults identifying as vegetarian (do not eat meat or fish) or vegan (do not eat any animal-sourced foods) in the National Diet and Nutrition Survey more than doubled from ~2% to ~5%, equivalent to around 3 million adults [1], and a YouGov poll in 2024 indicated that 13% of adults in the UK follow a flexitarian diet (mainly vegetarian, but occasionally consume meat and fish) [2]. Plant-based diets (e.g., flexitarian, vegetarian, and vegan) have the potential to substantially reduce the environmental impacts associated with the high consumption of meat and dairy products [3] and to benefit human health due to a generally lower consumption of saturated fat, an established risk factor for ischaemic heart disease [4]. However, plant-based diets may also increase the risk of nutrient deficiencies associated with avoiding animal-sourced foods (e.g., vitamin B12, calcium, and iodine), which can, in turn, lead to poor health outcomes [4]. However, evidence on the nutrient adequacy of plant-based diets is scarce and has been limited to vegans, vegetarians, and pescatarians and not considered flexitarian diet approaches [4]. Furthermore, the largest cohort studies including vegetarians and vegans were based on participants recruited in the 1990s and 2000s [5,6], when there was more limited availability of meat and dairy plant-based alternatives [7,8], which might have a specific contribution to nutrient intake (e.g., through fortified alternatives or supplementation).

Web-based studies have been demonstrated to be effective for collecting information on lifestyle and health from a diverse sample and have been used successfully to set up large cohort studies [9,10]. Several studies have demonstrated that online tools for dietary assessment perform well against traditional methods [11,12,13,14,15]. In the UK, a vast majority of adults use the Internet (92% or ~61.7 million users in 2020), including nearly all adults aged 16–44 and more than half aged 75 and over [16]. Using a web-based approach, we set up Feeding the Future (FEED), an online study collecting up-to-date information on plant-based diets (including information on contemporary meat and dairy alternatives), diets containing meat and fish, and associated personal characteristics in UK adults. Here, we describe the baseline characteristics of FEED participants and compare the mean daily nutrient intakes between diet groups and with recommended targets.

## 2. Materials and Methods

### 2.1. Recruitment

This study was open to UK residents aged 18 or over. Participants were recruited via various UK institutions and societies (e.g., the Vegan Society, the Vegetarian Society), mainstream broadcasters (e.g., radio, regional newsletters), food distributors (e.g., Waitrose weekend magazine), public engagement initiatives, social media, word of mouth, and posters displayed in local food/vegan markets and festivals with a brief summary and a QR code directing participants to the study site. A combination of generic and targeted advertising material (calls for participants from specific diet groups) was used. Informed consent was obtained electronically. An example ad can be seen in Appendix A.

### 2.2. Survey

The FEED survey comprised a one-off 20 min online questionnaire administered through JISC Online Surveys version 2 (https://www.ceu.ox.ac.uk/research/feeding-the-future-study-feed, accessed on 25 March 2024). Information was collected on dietary motivations, anthropometrics, sociodemographic and lifestyle information, and dietary intakes. The FEED online survey questions on lifestyle, health, and sociodemographic characteristics are aligned with other UK-based cohorts such as UK Biobank. Participants were asked to select a diet group that best described their current diet and report on the length of time that they have been following the diet. Diet group options included omnivore (consume meat frequently), flexitarian (infrequent meat eating), pescatarian (consume fish but no meat), vegetarian (do not consume meat or fish), and vegan (do not consume any animal-sourced foods). Height and weight were self-reported and subsequently used to calculate body mass index (BMI; weight in kilograms divided by height in metres squared). Additional questions on lifestyle characteristics included self-reported overall health status, smoking status, activity levels, supplement use, education level, and employment.

The FEED food frequency questionnaire (FFQ), included in the survey, was adapted from the validated UK European Prospective Investigation into Cancer and Nutrition (EPIC) study FFQ [17,18,19], and has been used in EPIC-Oxford, EPIC-Norfolk, Whitehall II, the Fenland Study, and the UK Women’s Cohort Study (with some modifications). The key features of the new online questionnaire are updates to contemporary foods and inclusion of a much wider range of plant-based meat alternative products and plant-based dairy alternative products, with questions to determine fortification with nutrients. The FEED study FFQ includes eight additional options for plant-based milk alternatives (soya, rice, oat, pea, almond, coconut, hemp, other), other vegan dairy alternatives (cream, yogurt, and ice cream), vegan eggs, options for vegan dips and sauces (tahini, vegan mayonnaise and salad cream/dressing), six meat alternatives (tempeh, textured vegetable protein, falafel, veggie burgers/sausages, soy burgers/sausages, seitan), and additional items such as chickpeas, oils, chocolate spread, nut/fruit bar, protein supplements (powders and bars), and meal replacements (shakes and bars). More detailed information on the types, brands, and fortification specifications of any plant-based alternatives consumed was collected through free text.

### 2.3. Database

Food composition data were provided in 2023 from the UK nutrient databank (NDB) [20] used for the UK National Diet and Nutrition Survey (NDNS) for the calculation of nutrient intakes (extract version: UK_NDB_1.2, 2023) [21]. This database is largely the same as the UK Composition of Foods Integrated Dataset (CoFID) food composition tables [22] but includes a larger range of foods and processed foods, and contains no missing values.

Additional foods and beverages were incorporated into the food composition database where (1) there was a lack of representation for vegan products in the NDB for a particular survey item (e.g., vegan hot chocolate), or (2) if the item was entirely missing (e.g., certain plant-based milk alternatives, meat alternatives, protein bars and powders, meal replacements). The selection of additional foods was guided by free-text responses where brands were explicitly asked in the survey, and, if not explicitly asked, several different representative options were selected from the FoodDB, a large database containing mandatory nutritional data for products available in major UK online supermarkets (up to May 2022) [23]. The selection of food codes for specific survey items was adapted, where participants were able to specify consuming fortified or non-fortified versions of a food. Nutritional information obtained from the FoodDB was cross-checked against online retailers for accuracy and updated to account for any potential changes to product composition (September 2023). Detailed information on the process of how additional foods were selected, imputation of missing nutrient values, and mapping survey items to food codes in the database can be found in Appendix B.

### 2.4. Intakes

To estimate daily food intakes, the frequency of consumption of each food or beverage item was multiplied by a standard Oxford WebQ portion size [24]; to estimate nutrient intakes (including total energy), this was further multiplied by the nutrient content. Where Oxford WebQ portion sizes were not available, portion sizes were obtained from EPIC-Oxford and UK Ministry of Agriculture, Fisheries and Food data [25]. For any additional plant-based alternative survey foods not listed in the above sources, portion sizes and nutritional data were obtained from product manufacturer websites, if not available in the NDB or FoodDB.

### 2.5. Dietary and Nutritional Guidelines

The Reference Nutrient Intake (RNI) and Estimated Average Requirement (EAR) values used to assess compliance with dietary guidelines were primarily based on the UK dietary reference values and proxy EARs developed by the Food and Nutrition Board of the US Institute of Medicine (IOM) for nutrients without established UK EARs [26,27,28,29]. Updated targets for free sugars and fibre (defined as recommended by the Association of Official Analytical Chemists International (AOAC)) were obtained from the Scientific Advisory Committee on Nutrition (SACN) Carbohydrate and Health (2015) report and the Salt and Health (2003) report for sodium [30,31]. The EAR cut-point method was used to estimate the prevalence of insufficient intakes of essential nutrients from dietary sources alone [32]. Additional alternative EAR values are provided for iron and zinc due to the suggested reduced bioavailability of these minerals in vegetarian and vegan diets [28]. Iron intake recommendations are conditional on age and gender, with a separate recommendation used for women aged 18–49.

### 2.6. Analysis

Descriptive statistics were used to summarise participant characteristics; results are reported as means with standard deviations (SDs), or counts and percentages, as appropriate. We grouped survey food items into major food categories and categories of plant-based sources (details in Supplementary Table S1), calculated mean daily intakes (in grams) for each diet group for women, men, and overall, and standardised to an intake of 2000 kcal. We calculated mean daily nutrient intakes in each diet group in women, men, and overall, adjusted for age and gender, and compared these to the guidelines mentioned above. As a sensitivity analysis, mean daily nutrient intakes were also calculated after taking into account vitamin, mineral, and supplement use in addition to food and drink, using available information. Doses were assumed based on products available in the NDB, and vitamin B12 injections were excluded due to the lack of corresponding food codes.

Analysis of variance (ANOVA) was used to assess overall differences in mean daily dietary and nutrient intakes between the five diet groups. Multiple pairwise comparisons with Bonferroni correction were used to assess the statistical significance of variations in adjusted mean nutrient intakes between different diet group pairs. *p*-Values < 0.05 were considered statistically significant. All analyses were conducted using Stata/SE 18.

## 3. Results

A total of 6684 participants were recruited into the FEED study between February 2022 and December 2023 from across the UK. Of these, 342 were excluded due to having implausible dietary intakes, defined as having an estimated daily energy intake (EI) of less than 3349 kJ (800 kcal) or more than 16,747 kJ (4000 kcal) for men (*n* = 75 men) and less than 2093 kJ (500 kcal) or more than 14,654 kJ (3500 kcal) for women (*n* = 240 women) [33], or where more than 20% of relevant FFQ items for each diet group (i.e., foods not excluded from that particular diet) were missing (*n* = 27).

Participant characteristics are presented in Table 1 overall and by diet group. Of 6342 participants with reliable data, 88.3% were from England, 4.5% from Wales, 6.4% from Scotland, and 0.9% from Northern Ireland. The survey was completed by 5116 women, 1126 men, and 100 adults who preferred not to state their gender, and respondents were primarily of white ethnicity (94.4%). The cohort comprised 1562 self-defined omnivores, 1349 flexitarians, 568 pescatarians, 1292 vegetarians, and 1571 vegans. The mean age (SD) of participants was 51.5 (15.9) years, with the youngest mean age reported by vegans 48.1 (15.2). Participants were mostly never-smokers (62.4%) and reported being in good or excellent health (78.8%). The cohort was mostly physically active (78.7%). Over three quarters of participants held a university degree. At the time of completing the survey, over half had a paid job, and a third stated having an annual household income of GBP 50 k or above. The majority of participants (ranging from 56.8% of vegans to 86.8% of vegetarians) reported following their current diet for 5+ years.

**Figure 1:**
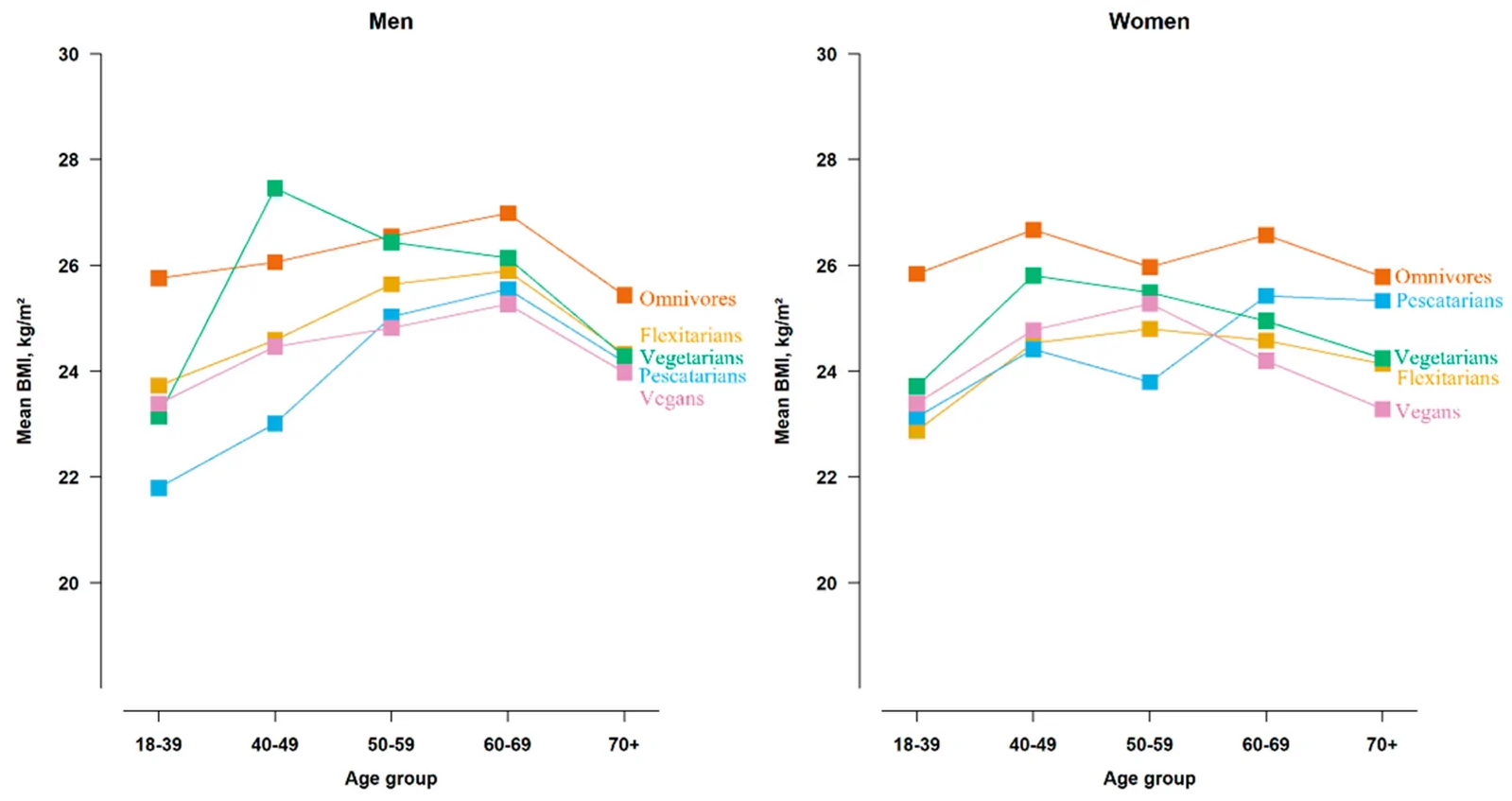
Mean body mass index (BMI) by age and diet group. Exclusions: 319 participants with missing values for height and weight, 100 participants who preferred not to state their gender.

Large differences in BMI were observed across diet groups; BMI was highest in omnivores (26.2 kg/m^2^), followed by vegetarians (24.9 kg/m^2^) and pescatarians (24.4 kg/m^2^), and lowest in the flexitarians and vegans (24.2 kg/m^2^ and 24.3 kg/m^2^, respectively). Omnivores had the highest proportion of obese adults of all diet groups (16.8%). Figure 1 shows mean BMI by diet and age group. In women of all ages, BMI was consistently highest in the omnivore group. Omnivorous and vegetarian men generally had higher BMI compared to flexitarians, pescatarians, and vegans in early to mid-life, with an increase seen between age groups 50–59 and 60–69 across all diets.

Absolute mean intakes of major food groups are presented in Table 2, and mean intakes standardised to 2000 kcal in Supplementary Table S2a,b. Significant differences in the amounts consumed by diet group were observed for all foods, with the exception of meal replacements in women, for which consumption was generally low and comparable across diet groups. Intakes for genders combined are presented in Supplementary Table S3. Overall mean consumption of meat (including poultry) was more than double in omnivores compared to flexitarians (137.9 g/day compared to 46.9 g/day). There was higher consumption of plant-based meat alternatives in groups that consumed less meat: 76.4 g/day in vegans compared to 10.9 g/day in omnivores. Egg consumption was highest in omnivores, with about one egg per day. Milk consumption was highest in omnivores and flexitarians, whereas consumption of plant-based milk alternatives was lowest in these groups. Although milk alternatives were consumed by all diet groups, vegans had the highest intakes (226.5 mL/day). Dairy-based cheese and yogurt intakes were similar across omnivores, flexitarians, pescatarians, and vegetarians. Vegan cheese and yogurt were consumed mostly by vegans. For both men and women, the intakes of grains, vegetables, and fruit were lowest in omnivores and highest in vegans. The consumption of nuts and seeds as well as pulses was highest in vegans, with vegans consuming more than double the amount of pulses compared to omnivores (110 g/day compared to 35.4 g/day). Few participants reported eating some items outside of their diet group restrictions [e.g., eggs (<2 g/day), dairy milk (<2 g/day), meat and fish (<1 g/day) in those describing their diet as vegan].

Table 3 shows the mean daily nutrient intakes by diet group adjusted for age and gender, and Supplementary Table S4a,b show these separately for women and men. Mean nutrient intakes differed across the diet groups (*p* < 0.001 for all nutrients). Energy intakes were highest in omnivores and flexitarians and lowest in vegetarians and vegans. The prevalence of under-reporting intakes was low to moderate across all diet groups, with ~20% of participants having an energy intake to basal metabolic rate ratio (EI/BMR) of less than 1.2. Protein consumption decreased with lower consumption of animal-sourced foods, with omnivores consuming ~6% more energy from protein than vegans (17.8% vs. 12.2%). Total fat contributed to around 35% of EI for all dietary groups, except for omnivores, where it contributed 40.4%. Fat composition also differed between the diet groups; the mean contribution of saturated fat to EI in omnivores was nearly double that of vegans (14.2% vs. 7.9%), while the polyunsaturated-fat-to-saturated-fat ratio was much lower in omnivores (0.5 vs. 1.21). Long-chain *n*-3 fatty acid intakes were comparable for all diet groups with the exception of vegetarians, who had a lower intake, whereas *n*-6 fatty acid intakes increased with the omission of animal-based foods, and trans fats decreased. Cholesterol intakes were highest in the groups that consumed more animal-based foods. Mean fibre intakes were relatively high across all diet groups, with vegans consuming around 18 g more per day compared to omnivores. The mean proportion of energy from carbohydrates was lowest in omnivores and highest in vegetarians and vegans. Free sugars made up more than 5% of EI (between 7% and 9%) in all diet groups.

Omnivores had the highest intakes of vitamin A and retinol and consumed three times more vitamin B12 than vegans. Thiamin, biotin, folate, beta-carotene, vitamin C, and vitamin E intakes were highest in vegans and lowest in omnivores. Vegetarians and vegans had significantly lower intakes of vitamin D compared with omnivores, flexitarians, and pescatarians. Intakes of niacin equivalents, riboflavin, and pantothenic acid differed significantly between the groups, with intakes generally decreasing with increased exclusion of animal-sourced foods. There was slight variation in vitamin B6 between the diet groups, with highest intakes among omnivores and lowest intakes in vegetarians. Sodium intake was highest in omnivores compared to other diet groups, whereas potassium was lowest in omnivores and vegetarians. Vegetarians and vegans had the lowest mean intakes of iodine and selenium, with iodine intakes being particularly low in vegans. Conversely, vegans had the highest intakes of iron and manganese. Calcium and magnesium intakes were lowest in omnivores. Some differences were observed between the groups for phosphorus and zinc intakes, with omnivores having higher intakes.

An increasing proportion of vitamin, mineral, and supplement use was seen with the consumption of fewer animal-sourced foods in the diet, with vegans reporting highest use (87.5%). Around 50% of vegans and 35% of vegetarians reported taking multivitamins combined with minerals. Vitamin B12 supplementation—most commonly in tablet form—was highest in vegans (~35–40%), followed by vegetarians and pescatarians (~20%), and then flexitarians and omnivores (~5–10%). Between 30% and 40% of participants across all diet groups reported taking vitamin D supplements. Iodine was taken predominantly by vegans, with ~8–12% compared to 2–3% in omnivores. Around a quarter of vegans took long-chain n-3 fatty acid supplements. Details on types of supplement use by diet group in men and women can be found in Supplementary Table S5a,b. Incorporating vitamin, mineral, and supplement use responses into nutrient calculations did not have a large impact on overall nutrient intakes for each diet group; the results of this are shown in Supplementary Table S6.

**Figure 2:**
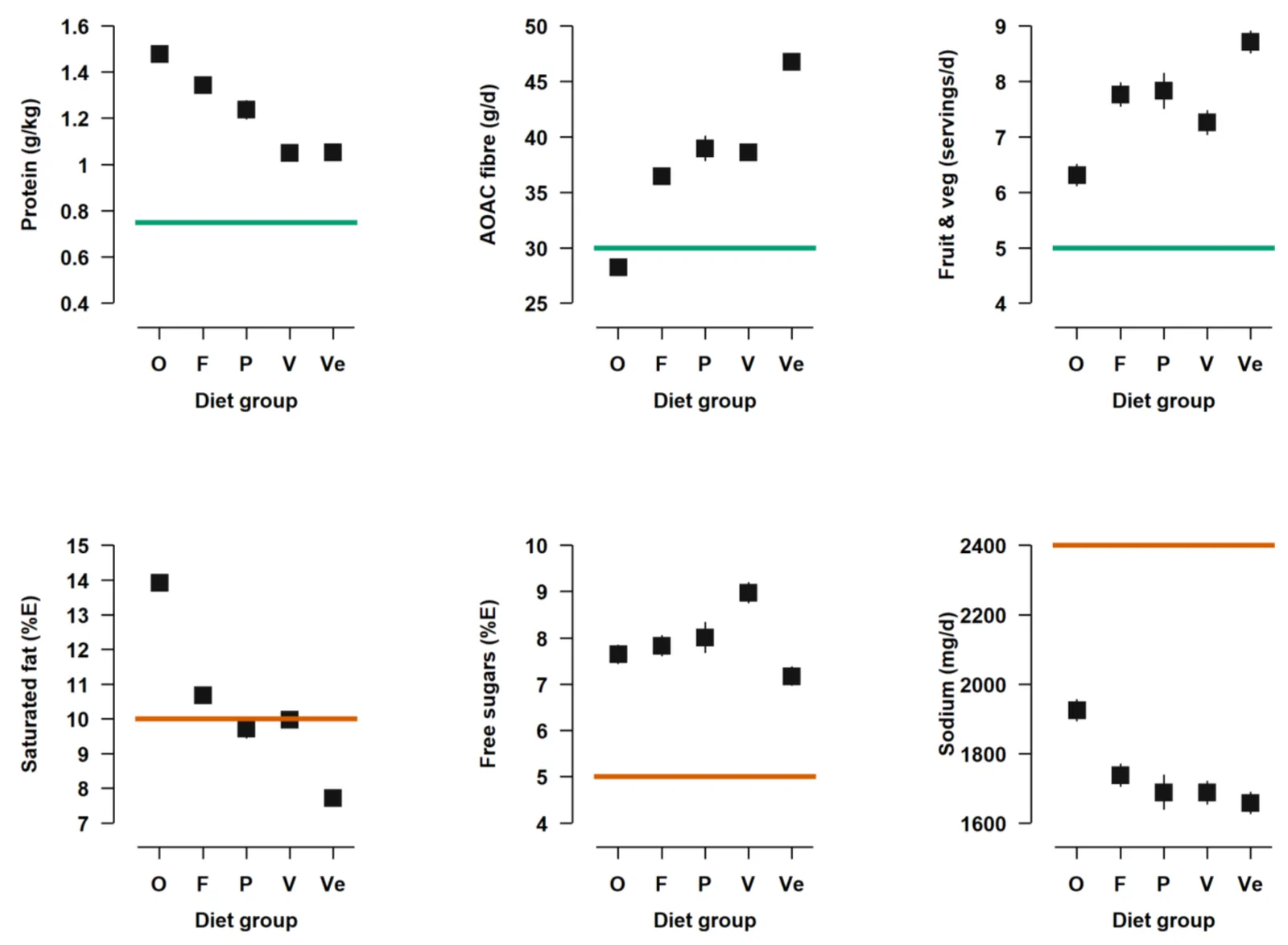
Daily dietary intakes in relation to UK dietary guidelines. Diet groups: O = omnivores, F = flexitarians, P = pescatarians, V = vegetarians, Ve = vegans; Black squares represent mean daily intakes and black lines represent standard errors; Reference lines represent the UK dietary recommendations for adults and reference nutrient intake (RNI) values for protein (green line = min, orange line = max).

Figure 2 presents the mean dietary intakes by diet group in relation to the UK population dietary guidelines, presented as RNI for protein. All diet groups met the daily recommendations for sodium (<2400 mg/d) and for fruit and vegetable intake (at least five portions per day), with higher intakes observed in diets containing fewer animal-sourced foods. The average intake of fibre in all diet groups, except for omnivores, met the population recommendation (≥30 g/d). All diet groups met the daily target for protein intake (0.75 g/kg of body weight), but lower values were observed for vegetarians and vegans. The average intake across all participants exceeded the recommendation for the maximum intake of free sugars (<5% energy). Vegans were the only group that met the saturated fat recommendation (<10% energy).

**Figure 3:**
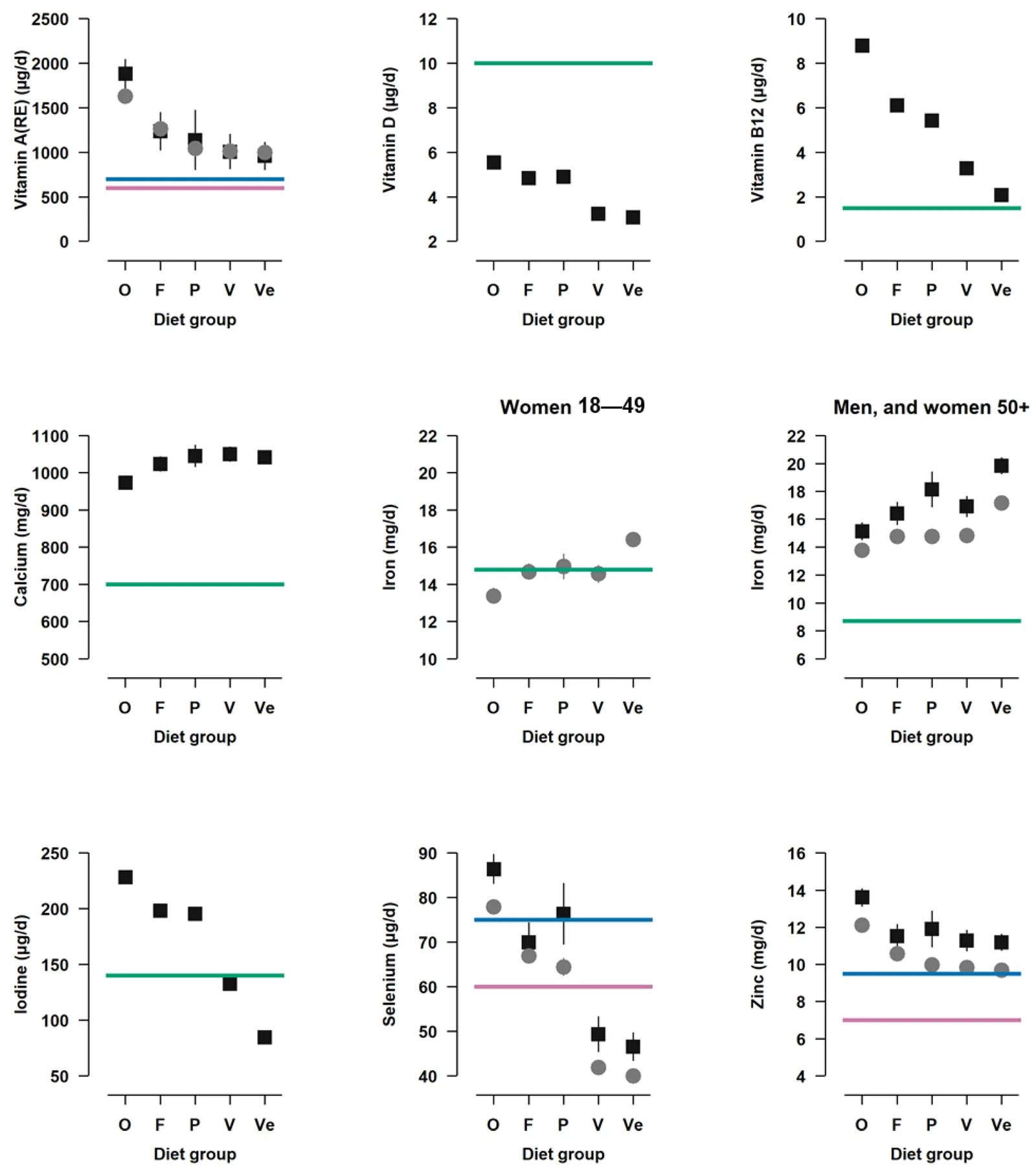
Daily micronutrient intakes in relation to UK Reference Nutrient Intakes (RNIs). Diet groups: O = omnivores, F = flexitarians, P = pescatarians, V = vegetarians, Ve = vegans; black squares represent mean daily intakes and black lines represent standard errors; intakes of vitamin A, iron, selenium, and zinc are presented separately by women (grey circles, grey standard error lines) and men (black squares, black standard error lines); reference lines represent the UK RNI for adults (green line) and sex-specific RNI targets for women (pink line) and men (blue line).

Mean daily intakes of micronutrients by diet group are presented against RNIs in Figure 3. All diet groups met the calcium recommendation. The RNIs for vitamin A, vitamin B12, and zinc were also met for all groups, with the lowest mean intakes observed in diets containing fewer animal-sourced foods. Women over 50 and men met the RNI for iron, whereas, in women aged 18–49, only vegans met the recommendation. Vegetarians and vegans had lower intakes of iodine and selenium compared to other diet groups. None of the diet groups met the recommendation for vitamin D.

Table 4 shows the proportion of women and men in each diet group who did not meet the recommended dietary targets. Some inadequacy for protein was observed in vegetarians and vegans (~10–15%). A degree of inadequacy for vitamin A intake was seen in the cohort, particularly for vegan men (19.3% below the target). Approximately one-third of vegan men and women did not meet the EARs for vitamin B12. Iron inadequacy was very low in all diet groups; however, after adjusting for bioavailability, some inadequacy was observed in vegetarians and vegans. Vegetarians and vegans also showed some inadequacy for zinc, and the proportion of women and men not meeting the target increased to ~30–50% after taking into account bioavailability. Over 50% of vegetarians and vegans did not meet the daily recommendation for selenium. The majority of vegans (~65%) and approximately one-quarter of vegetarians fell below the target for iodine intake.

## 4. Discussion

In launching the FEED study, we were successful in establishing the largest online cohort of UK adults consuming contemporary plant-based diets (e.g., flexitarian, vegetarian, and vegan), with complete dietary data available for 1571 vegans (~25% of the cohort). FEED is one of the largest studies of vegans in the world following the Adventist Health Study 2 (5548 vegans) and EPIC-Oxford (2596 vegans) cohorts, which were recruited in the 2000s and 1990s, respectively [5,6]. Our targeted recruitment strategies, including social media campaigns and virtual networks of various societies and institutions, proved to be an effective approach for recruiting participants following different plant-based diets from across all regions in the UK.

Participants recruited into the FEED study were mostly women, on average middle-aged, and the majority reported good health behaviours. Ages were similar across all diet groups except for vegans, who were around 4–5 years younger. Mean BMI differed substantially between the diet groups, with omnivores having the highest mean BMI, followed by vegetarians and pescatarians at 1–2 kg/m^2^ lower. The mean BMI in vegans and flexitarians was 2.5 kg/m^2^ lower than that of omnivores. Previous research has consistently shown that, in studies of people predominantly of white European ancestry living in high-income countries, meat-eaters have a higher BMI than non-meat eaters [6,34,35]. This is broadly consistent with our findings, with the exception of flexitarians, who had a mean BMI that was more comparable to that of non-meat eaters than omnivores. However, FEED participants were on average more overweight than what has been previously reported in UK studies with a large number of vegetarians [36,37]. The true prevalence of overweight is likely to be even higher given that self-reported measures of height and weight can lead to an underestimation of BMI by about 1 unit [38,39].

We found that the mean daily consumption of grains, vegetables, fruit, nuts/seeds, and pulses was lowest in omnivores and highest in vegans, and the consumption of plant-based alternatives was highest among participants who consumed fewer animal products. Similar trends have been shown in UK populations (EPIC-Oxford and UK Biobank participants), although daily intakes of plant-based meat alternatives and plant-based dairy alternatives were higher in the FEED cohort for all diet groups than previously reported [35,40]. The higher consumption of plant-based alternatives reported by our participants is in line with trends observed in the UK market, suggesting a larger contribution of these products to current overall diet and nutrient intakes [7,8].

We found a significant distinction in the frequency of meat consumption and meat alternative consumption between the two self-defined meat-eating groups in our study. The mean daily intake of total meat (including poultry) was relatively high in omnivores, and around double that of flexitarians, while the mean intake of plant-based meat alternatives in flexitarians was nearly threefold that consumed by omnivores. This suggests that the self-defined flexitarians in our study represent a unique group of omnivorous individuals who do in fact reduce their meat intake and increase their intake of alternatives. The omnivore group in our study reported eating more meat than ‘meat-eaters’ in previous UK studies comparing dietary intakes between diet groups, probably because we categorised flexitarians into a separate group [35,40], and the amount of meat consumed by flexitarians in our study was more similar to that of low meat-eaters in other studies [35,40,41]. Some within-group variation has been shown in studies on flexitarians, such as men consuming more meat and having more heterogeneous meat preferences; therefore, the types of meat consumed and the extent of substitution with plant-based alternatives should be further explored to better understand the dietary patterns of this group [42,43].

The most striking differences in nutrient intakes were observed between people consuming omnivorous and vegan diets, with flexitarians, pescatarians, and vegetarians generally falling in between. Vegans had the highest intakes of dietary fibre, polyunsaturated fatty acids, vitamin C, and vitamin E, while omnivores had the highest intakes of protein, saturated fatty acids, vitamin B12, vitamin D, and iodine. These findings are consistent with trends observed in the AHS-2 and EPIC-Oxford vegetarian cohorts [44,45]. A systematic review of observational and intervention studies from Europe, South/East Asia, and North America found similar patterns when assessing nutrient intakes and adequacy status in adult populations consuming plant-based diets, largely vegetarians and vegans, compared to those of meat-eaters [46].

Overall, participants met most of the dietary goals. AOAC fibre and fruit and vegetable intakes were generally high in the cohort compared to the national average [47] and recommendations; however omnivores fell just short of the daily fibre recommendation by 1.6 g. All diet groups in the cohort exceeded the UK recommendation for intake of free sugars (no more than 5% energy) by roughly 2–3%, and vegetarians by 4%, a similar pattern to that observed in EPIC-Oxford [44]. Interestingly, the mean intake of sodium was well below the recommended target in all diet groups and much lower than that reported by EPIC-Oxford participants [44], although it is important to note that the survey did not ask about added salt, which could lead to an underestimation of actual intakes. Vegans were the only diet group with saturated fat intakes meeting the recommended amount (7.9%, target < 10% energy). Pescatarians were borderline, with 9.9% of their EI coming from saturated fatty acids, and vegetarians, flexitarians, and omnivores exceeded the recommendation by 0.2%, 0.9%, and 4.2%, respectively. The pattern for saturated fat intakes by diet is similar to that reported in EPIC-Oxford participants, but the disparity between vegans and omnivores is even more pronounced in the FEED cohort [44].

Nutrients of possible concern for vegans typically include protein, vitamin B12, vitamin D, calcium, iodine, iron, selenium, and zinc [46,48]. Among FEED participants, protein intake was fairly high on average, with some inadequacy observed across the diet groups (<5% of omnivores and flexitarians, and 10–15% of vegans and vegetarians not meeting the EAR). It is possible that inadequacy in vegans could be higher when considering protein quality [26]. All diet groups met the vitamin B12 intake on average, but with some evidence of inadequacy among vegan participants. Overall, dietary intakes of vitamin B12 for vegans in our cohort were much higher than previously reported [44]. Similarly, calcium intakes were comparable among all diet groups, well above the recommended EARs, and markedly higher than previously reported in vegans. Vitamin D and iodine intakes were slightly higher overall, and, for vegans, higher than previously reported; however, none of the diet groups met the target for vitamin D and a high proportion of vegetarians and vegans did not meet the EAR for iodine. Selenium intakes were low and a high proportion of vegetarians and vegans did not meet the recommendation, consistent with previous findings [44,49].

We found a low prevalence of zinc and iron inadequacy in all diet groups and when restricted to older women, but, after adjusting for bioavailability, we saw a moderate to high estimated prevalence of inadequacy in vegetarians and vegans; this could relate to many plant-based foods (nuts and seeds, grains, pulses) containing a high phytate content, which may inhibit the absorption of zinc and iron [50,51]. Additionally, although the mean total iron intake was high in vegans, the predominant contributor was non-haem iron, which is less bioavailable than haem iron [28].

Vitamin D intake was well below the recommended target in all diet groups. Few foods naturally contain vitamin D; supplementation and ultraviolet B exposure may be required to achieve adequate levels [52,53]. Similar findings (inadequate intakes across all diet groups) for vitamin D have been reported for the EPIC-Oxford cohort, with lower plasma 25(OH)D concentrations in vegetarians and vegans compared to meat-eaters [44,54]. However, overall reported vitamin D intakes were higher in FEED, which may relate in part to more fortification in the plant-based milk alternatives.

The amount of plant-based milk consumed in our cohort was particularly high (~230 mL/day in vegans), and the majority of plant-based dairy alternatives reported by participants and contained in the NDB were fortified with calcium, and very frequently with vitamin B12 and vitamin D. This is broadly consistent with research that surveyed all plant-based dairy alternatives available in major UK supermarkets [55]. Due to widespread fortification of plant-based milk with calcium, several studies have reported no differences in the calcium content between dairy milk and plant-based milk alternatives [55,56]. However, there are still some uncertainties regarding bioavailability as evidence on calcium absorption from calcium added to plant-based beverages is limited to soy-based products [57]. The absorption of calcium can vary according to the type of added fortificant, and circulating concentrations of calcium are highly regulated in the body so it is difficult to assess differences in absorption from different sources [58,59]. Vitamin B12 intake from milk substitutes has been found to be a significant contributor to plasma vitamin B12 concentration, and if fortified products are consumed, the vitamin B12 levels could meet RNI targets [46,55,60].

Fortification of plant-based dairy alternatives with iodine, however, is not widespread in the UK, and the natural concentration of iodine in these products is low [61]. Dairy products are an important source of iodine in the UK population (mainly due to the fortification of cattle feed and the use of iodine-containing teat disinfectants), and individuals who consume plant-based dairy alternatives instead of dairy products are therefore at risk of iodine deficiency [62,63]. A study surveying plant-based dairy alternative products available on the UK market in 2023 found that only 28% of milk alternatives were fortified with iodine, compared to 88% with calcium and 83% with vitamin B12, and the figure was even lower for other dairy alternatives such as plant-based yogurt and cheese [56]. We observed some fortification of plant-based meat alternative products as reported by participants in the free text; the majority of alternatives consumed were fortified with vitamin B12 and iron, and none were additionally fortified with zinc. This is less likely to have a major contribution to overall nutrient intake as daily intakes of meat alternatives were generally lower compared to dairy alternatives.

UK legislation mandates fortification of bread and flour with calcium, iron, niacin, and thiamin (with folic acid to be additionally introduced in the near future), and margarine with vitamins A and D, though fortification of spreadable fats not legally classified as margarine is voluntary [64,65]. Voluntary fortification has the potential to make a meaningful contribution to intakes of vitamins and minerals for frequently consumed foods, which may explain the higher intakes of some nutrients in our cohort [66].

We set up the largest and most up-to-date online cohort of UK adults consuming plant-based diets and diets containing meat and fish. Our study used an adapted EPIC-Oxford FFQ, including additional items and a wider range of plant-based alternatives, allowing for better representation of foods consumed as part of contemporary diets. Obtaining fortification specifications for the top-consumed brands from free text also facilitated accounting for any differences in product fortification, and thus allowed for a more accurate estimation of nutrients at the individual and diet-group level. However, several potential limitations of our study should be noted. The FFQ was designed to evaluate a participant’s dietary intake over the previous year, whereas our questionnaire asked participants to report on their current diet even if they have been following it for a short time (e.g., Veganuary), limiting the ability to distinguish between recent and long-term dietary habits; however, only a small proportion of participants (ranging from 1.8% of vegetarians to 6.7% of vegans) reported following their current diet for less than one year. Another potential limitation of the study is that the survey did not assess the frequency of consumption for plant-based egg alternatives, though only ~5% of participants reported consuming these. The use of vitamins, minerals, and other supplements has not been factored into our nutrient calculations in the main analysis, resulting in a possible underestimation of some nutrients. This could have important implications for assessing nutrient adequacy, especially in instances where mean daily intakes did not meet UK dietary recommendations but reported supplementation was high among participants; for example, the reported use of vitamin D in all diet groups and vitamin B12 in vegans was as high as 30–40%. Due to the lack of information on the dose and specific products/brands, quantitative data on the intakes of vitamins, minerals, and supplements in FEED participants are limited. Sensitivity analysis calculations that take reported vitamin, mineral, and supplement use into account suggest that supplement use has a small impact on overall nutrient intakes, though additional information on the actual consumed dose is needed. Our study highlights the importance of the detailed collection of supplement consumption in studies of participants following plant-based diets.

We attempted to reduce the spurious assignment of traces of nutrients only found in animal foods to vegans by using plant-based alternatives in nutrient calculations where possible, though this could not be entirely avoided due to assumptions made when coding generic FFQ items and possible errors in reporting. However, the low daily intakes of animal-sourced food groups reported by vegans suggest that misreporting and/or occasional consumption of animal foods by vegans was minimal. The low prevalence of participants with an EI/BMR ratio of less than 1.2 (representing physiologically implausible energy values in relation to gender and body weight) suggests that, although some under-reporting of energy intakes may be present in the cohort, the FFQ performs reasonably well overall and across all diet groups. Additionally, due to potential measurement error, it is possible that we overestimated the proportion of people in each diet group with nutrient intakes below the recommendations. Despite efforts to recruit a representative sample of UK adults through using a web-based approach, the cohort still lacks in diversity; the cohort comprises mainly women of white ethnicity, and thus findings may not be generalisable to the total UK population of plant-based eaters. In addition, with the large proportion of women making up the FEED cohort, it is possible that other sources of reporting bias (e.g., social desirability bias) may be present, potentially leading to an underestimation of energy intakes in this cohort [67].

## 5. Conclusions

We set up the largest online cohort to date of plant-based diets in the UK, compared personal characteristics and dietary intakes between adults following five diet groups, and assessed their diets with reference to dietary guidelines. We observed large differences in dietary intakes between diet groups, with increased consumption of meat and dairy alternatives as animal-sourced food intakes decreased, particularly for plant-based milk alternatives. Notably, flexitarians consumed less than half the amount of meat and three times the amount of meat alternatives compared to omnivores, and the consumption of plant-based milk was particularly high in vegans. Our results show that the great majority of participants met the UK dietary recommendations for fruit and vegetables, sodium, and protein, though vegetarians and vegans had lower protein intakes. All diet groups (except omnivores) met the fibre recommendation, vegans were the only group that met the saturated fat recommendation, and all diet groups exceeded the target upper limit on free sugars. Calcium intake was similar across diet groups and well above the dietary recommendation, while average vitamin D intake was below the RNI in all groups. Our findings suggest possible inadequacies for some micronutrients (zinc, iodine, and selenium) among both vegetarians and vegans, and vitamins A and B12 among vegans. Overall FEED participants showed good compliance with most dietary recommendations. The risk for inadequate intakes of certain nutrients in people following contemporary plant-based diets should be considered and might be mitigated by improved dietary choices and/or food fortification and use of supplements.

## Figures and Tables

**Table 1:** Baseline characteristics of the FEED cohort by diet group.

	Omnivorous	Flexitarian	Pescatarian	Vegetarian	Vegan	All	*p*-Value
*N*	1562 (24.6%)	1349 (21.3%)	568 (9.0%)	1292 (20.4%)	1571 (24.8%)	6342 (100.0%)	
Length following diet							
<1 year	56 (3.6%)	90 (6.7%)	23 (4.0%)	23 (1.8%)	106 (6.7%)	298 (4.7%)	<0.001
1–4 years	161 (10.3%)	386 (28.6%)	138 (24.3%)	148 (11.5%)	572 (36.4%)	1405 (22.2%)	
5–19 years	154 (9.9%)	463 (34.3%)	154 (27.1%)	212 (16.4%)	648 (41.2%)	1631 (25.7%)	
20+ years	1191 (76.2%)	410 (30.4%)	253 (44.5%)	909 (70.4%)	245 (15.6%)	3008 (47.4%)	
Age (years)	52.8 ± 14.6	52.0 ± 16.9	53.3 ± 16.3	53.0 ± 16.2	48.1 ± 15.2	51.5 ± 15.9	<0.001
Gender							
Female	1243 (79.6%)	1153 (85.5%)	493 (86.8%)	1060 (82.0%)	1167 (74.3%)	5116 (80.7%)	
Male	310 (19.8%)	176 (13.0%)	71 (12.5%)	216 (16.7%)	353 (22.5%)	1126 (17.8%)	<0.001
Prefer not to say	9 (0.6%)	20 (1.5%)	4 (0.7%)	16 (1.2%)	51 (3.2%)	100 (1.6%)	
BMI (kg/m^2^)	26.2 ± 5.8	24.2 ± 4.9	24.4 ± 5.0	24.9 ± 5.4	24.3 ± 4.9	24.9 ± 5.3	<0.001
Ethnicity							
White	1468 (94.0%)	1254 (93.0%)	536 (94.4%)	1245 (96.4%)	1482 (94.3%)	5985 (94.4%)	0.012
Black	4 (0.3%)	10 (0.7%)	5 (0.9%)	3 (0.2%)	4 (0.3%)	26 (0.4%)	
Asian	39 (2.5%)	47 (3.5%)	12 (2.1%)	23 (1.8%)	32 (2.0%)	153 (2.4%)	
Other	28 (1.8%)	23 (1.7%)	11 (1.9%)	11 (0.9%)	30 (1.9%)	103 (1.6%)	
Do not want to disclose	23 (1.5%)	15 (1.1%)	4 (0.7%)	10 (0.8%)	23 (1.5%)	75 (1.2%)	
Overall health							
Good or excellent	1204 (77.1%)	1077 (79.8%)	456 (80.3%)	969 (75.0%)	1293 (82.3%)	4999 (78.8%)	<0.001
Fair	308 (19.7%)	232 (17.2%)	98 (17.3%)	258 (20.0%)	217 (13.8%)	1113 (17.5%)	
Poor	47 (3.0%)	38 (2.8%)	14 (2.5%)	59 (4.6%)	54 (3.4%)	212 (3.3%)	
Do not know/prefer not to say	3 (0.2%)	2 (0.1%)	0 (0.0%)	6 (0.5%)	7 (0.4%)	18 (0.3%)	
Ever smoked tobacco							
No, never	958 (61.3%)	886 (65.7%)	341 (60.0%)	827 (64.0%)	944 (60.1%)	3956 (62.4%)	0.004
Yes, in the past	524 (33.5%)	422 (31.3%)	202 (35.6%)	428 (33.1%)	558 (35.5%)	2134 (33.6%)	
Yes, now	80 (5.1%)	41 (3.0%)	25 (4.4%)	37 (2.9%)	69 (4.4%)	252 (4.0%)	
Vigorous exercise in the last 12 months							
No	340 (21.8%)	266 (19.7%)	115 (20.2%)	327 (25.3%)	301 (19.2%)	1349 (21.3%)	<0.001
Yes	1222 (78.2%)	1083 (80.3%)	453 (79.8%)	965 (74.7%)	1270 (80.8%)	4993 (78.7%)	
Highest attained education							
GCSEs *	127 (8.1%)	71 (5.3%)	37 (6.5%)	101 (7.8%)	121 (7.7%)	457 (7.2%)	<0.001
A levels ^Ɨ^	225 (14.4%)	155 (11.5%)	73 (12.9%)	221 (17.1%)	273 (17.4%)	947 (14.9%)	
University degree	1163 (74.5%)	1101 (81.6%)	441 (77.6%)	934 (72.3%)	1128 (71.8%)	4767 (75.2%)	
None	12 (0.8%)	7 (0.5%)	7 (1.2%)	16 (1.2%)	17 (1.1%)	59 (0.9%)	
Do not want to disclose	35 (2.2%)	15 (1.1%)	10 (1.8%)	20 (1.5%)	32 (2.0%)	112 (1.8%)	
Paid job at present							
Yes	961 (61.5%)	792 (58.7%)	332 (58.5%)	729 (56.4%)	1020 (64.9%)	3834 (60.5%)	<0.001
No	131 (8.4%)	108 (8.0%)	48 (8.5%)	127 (9.8%)	197 (12.5%)	611 (9.6%)	
Retired	429 (27.5%)	424 (31.4%)	178 (31.3%)	405 (31.3%)	300 (19.1%)	1736 (27.4%)	
Do not want to disclose	41 (2.6%)	25 (1.9%)	10 (1.8%)	31 (2.4%)	54 (3.4%)	161 (2.5%)	
Average total income before tax received by household							
<GBP 18,000-GBP 49,999	673 (43.1%)	624 (46.3%)	258 (45.4%)	586 (45.4%)	808 (51.4%)	2949 (46.5%)	<0.001
GBP 50,000–GBP 100,000	585 (37.5%)	474 (35.1%)	197 (34.7%)	396 (30.7%)	460 (29.3%)	2112 (33.3%)	
Do not want to disclose	304 (19.5%)	251 (18.6%)	113 (19.9%)	310 (24.0%)	303 (19.3%)	1281 (20.2%)	
Region							
England							
East Midlands	205 (13.2%)	130 (9.7%)	73 (12.9%)	161 (12.5%)	210 (13.4%)	779 (12.3%)	<0.001
Greater London	131 (8.4%)	207 (15.5%)	81 (14.3%)	157 (12.2%)	191 (12.2%)	767 (12.2%)	
North East	82 (5.3%)	55 (4.1%)	39 (6.9%)	88 (6.8%)	109 (7.0%)	373 (5.9%)	
North West	109 (7.0%)	92 (6.9%)	38 (6.7%)	131 (10.2%)	169 (10.8%)	539 (8.5%)	
South East	542 (34.9%)	442 (33.0%)	163 (28.8%)	320 (24.8%)	349 (22.3%)	1816 (28.8%)	
South West	189 (12.2%)	180 (13.5%)	78 (13.8%)	150 (11.6%)	217 (13.9%)	814 (12.9%)	
West Midlands	125 (8.0%)	94 (7.0%)	36 (6.4%)	117 (9.1%)	112 (7.2%)	484 (7.7%)	
Northern Ireland	12 (0.8%)	7 (0.5%)	3 (0.5%)	13 (1.0%)	19 (1.2%)	54 (0.9%)	
Scotland	93 (6.0%)	85 (6.4%)	29 (5.1%)	100 (7.8%)	95 (6.1%)	402 (6.4%)	
Wales	66 (4.2%)	46 (3.4%)	26 (4.6%)	52 (4.0%)	91 (5.8%)	281 (4.5%)	

Data are presented as mean ± SD for continuous measures, and *n* (%) for categorical measures. *p*-Values for ANOVA and Chi2 tests. * GCSE—qualification in a specific subject typically taken by school students aged 14–16 in the UK except Scotland. ^Ɨ^ A-Levels (Advanced Level qualifications)—UK subject-based qualification for students aged 16 and above (includes Scottish Highers).

**Table 2:** (a) Absolute mean intakes of major food groups in women. (b) Absolute mean intakes of major food groups in men.

**(a)**						
	**Omnivorous**	**Flexitarian**	**Pescatarian**	**Vegetarian**	**Vegan**	***p*-Value**
*N*	1243 (24.3%)	1153 (22.5%)	493 (9.6%)	1060 (20.7%)	1167 (22.8%)	
Meat (g/day)	132.6 ± 112.9	46.5 ± 29.8	1.0 ± 6.7	0.0 ± 0.7	0.1 ± 1.0	<0.001
Fish (g/day)	51.0 ± 29.8	48.8 ± 30.8	52.8 ± 33.0	1.0 ± 4.7	0.4 ± 3.8	<0.001
Plant-based meat alternatives (g/day)	10.9 ± 14.8	30.8 ± 30.5	50.3 ± 37.4	59.1 ± 40.4	73.3 ± 47.4	<0.001
Eggs (g/day)	46.0 ± 53.1	32.7 ± 39.1	37.2 ± 53.8	33.0 ± 47.9	0.6 ± 5.4	<0.001
Milk (mL/day)	188.0 ± 159.4	142.1 ± 146.6	114.9 ± 138.8	99.8 ± 132.2	0.6 ± 10.0	<0.001
Plant-based milk alternatives (mL/day)	36.2 ± 88.5	83.7 ± 121.6	112.9 ± 140.5	120.5 ± 141.9	225.9 ± 159.7	<0.001
Cheese (g/day)	20.7 ± 20.6	16.9 ± 14.2	16.4 ± 14.4	19.4 ± 19.0	0.1 ± 1.4	<0.001
Plant-based cheese alternatives (g/day)	0.5 ± 3.5	0.9 ± 2.7	1.6 ± 4.1	2.5 ± 4.8	8.3 ± 9.3	<0.001
Yogurt (g/day)	50.9 ± 54.0	55.9 ± 62.6	48.9 ± 54.6	42.6 ± 52.8	0.3 ± 4.7	<0.001
Plant-based yogurt alternatives (g/day)	2.7 ± 15.1	7.7 ± 23.8	9.4 ± 25.1	14.2 ± 31.1	37.0 ± 47.4	<0.001
Pulses (g/day)	35.8 ± 31.2	59.0 ± 42.0	70.1 ± 47.7	72.2 ± 44.2	104.5 ± 66.6	<0.001
Nuts/seeds (g/day)	16.3 ± 21.5	22.4 ± 23.4	23.8 ± 24.5	23.2 ± 23.4	34.0 ± 31.6	<0.001
Grains (g/day)	221.9 ± 143.4	284.8 ± 126.7	294.4 ± 124.9	289.4 ± 123.6	314.8 ± 133.0	<0.001
Vegetables (g/day)	353.1 ± 207.0	406.1 ± 215.4	415.6 ± 213.4	385.2 ± 208.6	459.6 ± 247.9	<0.001
Fruit (g/day)	185.4 ± 149.9	249.4 ± 170.2	234.8 ± 158.1	220.8 ± 148.1	254.5 ± 197.9	<0.001
Confectionery (g/day)	59.7 ± 50.1	62.8 ± 47.2	60.7 ± 46.3	69.1 ± 45.4	52.4 ± 37.9	<0.001
Protein shakes/bars (g/day)	1.6 ± 7.8	1.8 ± 7.9	1.7 ± 6.6	2.0 ± 8.5	3.7 ± 12.9	<0.001
Meal replacements (g/day)	1.0 ± 9.7	1.2 ± 12.4	0.6 ± 5.4	1.5 ± 11.7	2.0 ± 12.9	0.139
Tea/coffee (mL/day)	631.2 ± 331.7	631.9 ± 314.2	625.0 ± 303.8	593.3 ± 317.7	581.8 ± 327.7	<0.001
Non-alcoholic drinks (mL/day)	261.0 ± 365.6	194.2 ± 269.4	202.6 ± 292.8	249.1 ± 327.2	237.0 ± 310.6	<0.001
Alcoholic drinks (mL/day)	93.6 ± 135.1	97.6 ± 115.6	103.9 ± 133.3	96.4 ± 123.6	80.2 ± 121.6	0.001
**(b)**						
	**Omnivorous**	**Flexitarian**	**Pescatarian**	**Vegetarian**	**Vegan**	***p*** **-Value**
*N*	310 (27.5%)	176 (15.6%)	71 (6.3%)	216 (19.2%)	353 (31.3%)	
Meat (g/day)	158.9 ± 134.9	49.0 ± 30.1	2.6 ± 10.7	0.5 ± 4.6	0.2 ± 2.7	<0.001
Fish (g/day)	56.3 ± 69.6	48.7 ± 39.4	67.8 ± 56.0	0.7 ± 4.1	0.4 ± 3.5	<0.001
Plant-based meat alternatives (g/day)	10.7 ± 17.8	36.0 ± 35.6	53.1 ± 38.6	71.0 ± 48.8	84.1 ± 54.7	<0.001
Eggs (g/day)	55.4 ± 64.1	32.5 ± 39.5	39.1 ± 44.3	40.1 ± 62.7	1.4 ± 15.3	<0.001
Milk (mL/day)	232.0 ± 191.5	151.3 ± 160.3	124.6 ± 165.3	126.7 ± 141.8	1.9 ± 25.5	<0.001
Plant-based milk alternatives (mL/day)	24.4 ± 81.9	73.7 ± 120.9	113.0 ± 158.3	114.6 ± 156.5	233.1 ± 166.7	<0.001
Cheese (g/day)	21.7 ± 21.5	17.8 ± 17.1	19.6 ± 23.3	20.4 ± 17.5	0.1 ± 1.4	<0.001
Plant-based cheese alternatives (g/day)	0.3 ± 1.5	1.3 ± 4.7	1.6 ± 4.7	2.7 ± 4.8	8.5 ± 10.4	<0.001
Yogurt (g/day)	43.2 ± 51.3	51.3 ± 56.2	49.0 ± 75.8	44.8 ± 49.2	0.6 ± 7.5	<0.001
Plant-based yogurt alternatives (g/day)	1.5 ± 10.7	7.7 ± 23.3	8.4 ± 19.4	8.7 ± 19.0	32.3 ± 50.7	<0.001
Pulses (g/day)	33.6 ± 31.1	57.4 ± 46.0	92.5 ± 102.7	73.5 ± 44.4	126.6 ± 101.3	<0.001
Nuts/seeds (g/day)	15.1 ± 20.3	26.2 ± 29.0	27.6 ± 23.2	24.3 ± 24.9	40.9 ± 39.0	<0.001
Grains (g/day)	252.2 ± 165.2	322.0 ± 142.4	362.2 ± 182.8	348.0 ± 131.7	379.5 ± 180.8	<0.001
Vegetables (g/day)	291.4 ± 203.3	339.7 ± 160.7	413.2 ± 220.1	367.5 ± 185.6	433.5 ± 231.9	<0.001
Fruit (g/day)	183.0 ± 168.5	259.8 ± 166.3	280.5 ± 200.3	240.4 ± 183.3	291.0 ± 224.7	<0.001
Confectionery (g/day)	62.0 ± 58.1	62.3 ± 45.6	51.5 ± 36.1	68.2 ± 48.6	56.1 ± 50.2	0.037
Protein shakes/bars (g/day)	2.6 ± 9.9	4.7 ± 17.9	4.6 ± 17.1	1.8 ± 6.8	4.7 ± 13.2	0.031
Meal replacements (g/day)	2.2 ± 15.2	4.0 ± 27.7	1.4 ± 9.2	1.2 ± 10.5	7.2 ± 36.9	0.031
Tea/coffee (mL/day)	573.5 ± 346.0	625.9 ± 330.9	668.5 ± 295.6	640.9 ± 316.1	518.2 ± 346.6	<0.001
Non-alcoholic drinks (mL/day)	294.1 ± 428.2	187.3 ± 233.6	177.0 ± 248.9	265.5 ± 301.0	237.0 ± 338.1	0.004
Alcoholic drinks (mL/day)	204.2 ± 286.8	194.2 ± 232.9	295.4 ± 390.3	186.1 ± 247.4	141.8 ± 222.2	<0.001

ANOVA was used to compare the means between the diet groups; 100 participants who preferred not to state their gender were excluded.

**Table 3:** Daily dietary nutrient intakes, adjusted for age and gender.

	Omnivores	Flexitarians	Pescatarians	Vegetarians	Vegans
Energy (kJ)	9511 ^b^	9388 ^b^	9277 ^a,b^	9047 ^a^	9132 ^a^
Energy/BMR ratio	1.59 ^a,b^	1.63 ^b^	1.60 ^a,b^	1.55 ^a^	1.59 ^a^
Energy/BMR ratio < 1.2 (%)	18.8 ^a,b^	17.2 ^a^	19.1 ^a,b^	21.9 ^b^	21.1 ^a,b^
Carbohydrate (%E)	37.8	44.8	46.4	48.9 ^a^	49.2 ^a^
Total sugars (%E)	17.4	19.9 ^a^	20.1 ^a,b^	20.6 ^b^	20.2 ^a,b^
Free sugars (%E)	7.67 ^a,b^	7.85 ^b^	8.02 ^b^	8.99	7.40 ^a^
Starch (%E)	20.5	25.0	26.4	28.4	30.8
Protein (%E)	17.8	15.1	14.2	12.6	12.2
Protein (g) per kg body weight	1.49	1.34	1.24	1.06 ^a^	1.06 ^a^
Fat (%E)	40.4	36.2 ^b^	35.3 ^a,b^	34.8 ^a^	35.1 ^a^
SFA (%E)	14.2	10.9	9.9 ^a^	10.2 ^a^	7.9
MUFA (%E)	16.0	14.9 ^b^	14.7 ^a,b^	14.1 ^a^	14.9 ^b^
PUFA (%E)	6.5	7.0	7.4 ^a^	7.4 ^a^	9.2
P/S ratio	0.50	0.68	0.78 ^a^	0.76 ^a^	1.21
Cholesterol (mg)	415	248	217	168	20
*n*-3 fatty acids (g)	2.83 ^a^	2.80 ^a^	2.86 ^a^	2.35	2.83 ^a^
*n*-6 fatty acids (g)	13.8	15.1 ^a^	15.7 ^a,b^	15.9 ^b^	20.2
Trans fatty acids (g)	1.45	0.95 ^a^	0.77 ^a^	0.80	0.38
Alcohol (%E)	2.85 ^a^	2.85 ^a^	3.16 ^a^	2.72 ^a^	2.23
Alcohol (g)	9.38 ^a,b^	9.24 ^a,b^	10.26 ^b^	8.45 ^a^	6.98
AOAC Fibre (g)	28.4	36.7	39.3 ^a^	38.8 ^a^	46.7
β-Carotene (μg)	3668	4240 ^a^	4204 ^a^	4039 ^a^	4859
Retinol (μg)	997	466	272 ^a^	259 ^a^	102
Vitamin A (RE) (µg)	1682	1261	1057 ^a^	1009 ^a^	989 ^a^
Vitamin D (μg)	5.57	4.82 ^a^	4.82 ^a^	3.07	2.60
Thiamin (mg)	2.07	2.19 ^a^	2.23 ^a,b^	2.28 ^b^	2.62
Riboflavin (mg)	2.26	2.03 ^b^	1.96 ^a,b^	1.94 ^a^	1.80
Niacin equivalent (mg)	47.0	40.2	36.9	31.9	33.3
Vitamin C (mg)	151	170 ^a^	175 ^a^	167 ^a^	193
Vitamin E (mg)	14.1	15.7 ^a^	16.7 ^b^	16.0 ^a,b^	18.7
Vitamin B6 (mg)	2.30	2.15	2.05 ^a^	1.90	2.04 ^a^
Vitamin B12 (μg)	9.78	6.49	5.55	3.35	2.04
Folate (μg)	362	401 ^a^	413 ^a^	411 ^a^	453
Pantothenic acid (mg)	8.25	7.42	6.90	6.42	5.84
Biotin (µg)	56.2	59.4 ^a^	61.4 ^a^	59.2 ^a^	67.4
Sodium (mg)	1973	1816 ^b^	1769 ^a,b^	1761 ^a,b^	1712 ^a^
Potassium (mg)	3923 ^a^	4072 ^b^	4085 ^b^	3836 ^a^	4144 ^b^
Calcium (mg)	976	1031 ^a^	1054 ^a^	1055 ^a^	1022 ^a^
Magnesium (mg)	363	414 ^a^	430 ^a^	413 ^a^	478
Phosphorus (mg)	1619	1557 ^b^	1537 ^b^	1420 ^a^	1397 ^a^
Iron (mg)	13.9	15.1 ^a^	15.4 ^a^	15.2 ^a^	17.6
Haem iron (mg)	1.05	0.49	0.30	0.16	0.18 ^a^
Non-haem iron (mg)	12.8	14.5 ^a^	15.1 ^a,b^	15.1 ^b^	17.3
Copper (mg)	1.80 ^a^	1.86 ^a^	1.86 ^a^	1.81 ^a^	2.20
Zinc (mg)	12.4	10.8	10.3 ^a^	10.1 ^a^	10.0 ^a^
Chloride (mg)	3472	3269 ^a,b^	3177 ^a^	3166 ^a^	3363 ^b^
Iodine (μg)	232	203 ^a^	200 ^a^	135	89
Manganese (mg)	4.23	5.64	6.12 ^a^	6.14 ^a^	7.37
Selenium (μg)	79.7	67.5 ^b^	66.4 ^b^	43.2 ^a^	41.3 ^a^

Energy/BMR ratio—energy intake to basal metabolic rate ratio; SFAs—saturated fatty acids; PUFAs—polyunsaturated fatty acids; P/S ratio—polyunsaturated fat (g)/saturated fat (g); values are presented as means adjusted for age and gender. ANOVA was used to compare the means between the diet groups. Multiple pairwise comparisons with Bonferroni correction were used to determine the statistical significance of differences in intakes between pairs of diet groups. ^a,b^ Pairs of means in the same row sharing a common superscript are not significantly different at the 5% level. *p*-Values for heterogeneity between diet groups for all nutrients were less than 0.0001. *N* = 6342: 1562 omnivores, 1349 flexitarians, 568 pescatarians, 1292 vegetarians, and 1571 vegans.

**Table 4:** Prevalence of inadequate intakes by gender and diet group.

Nutrient	EAR Value ^a^	Omnivores		Flexitarians		Pescatarians		Vegetarians		Vegans	
		W	M	W	M	W	M	W	M	W	M
Protein	0.66 g/kg of body weight ^b^	1.6%	3.3%	3.1%	5.8%	5.5%	5.8%	11.6%	10.7%	13.7%	15.0%
Vitamin A (RE)	M: 500 µg, W: 400 µg	1.2%	4.2%	1.5%	5.1%	2.6%	8.5%	4.8%	8.3%	8.9%	19.3%
Thiamin	0.3 mg per 1000 kcal energy	0.0%	0.0%	0.0%	0.0%	0.0%	0.0%	0.0%	0.0%	0.0%	0.0%
Riboflavin	M: 1.0 mg, W: 0.9 mg	0.6%	1.9%	1.7%	2.8%	3.4%	1.4%	2.7%	1.4%	5.6%	7.4%
Niacin	5.5 mg per 1000 kcal energy	0.0%	0.0%	0.0%	0.0%	0.0%	0.0%	0.0%	0.0%	0.0%	0.0%
Vitamin B6	13 µg per g protein	0.9%	1.3%	0.3%	0.0%	0.0%	0.0%	0.0%	0.0%	0.3%	0.3%
Vitamin B12	1.25 µg	0.1%	0.0%	0.8%	0.0%	0.2%	1.4%	4.7%	3.2%	31.2%	30.0%
Folate	150 µg	3.7%	2.9%	0.6%	0.0%	0.6%	0.0%	0.8%	0.0%	0.8%	0.0%
Vitamin C	25 mg	4.1%	6.1%	0.2%	0.0%	0.2%	0.0%	0.0%	0.0%	0.1%	0.0%
Calcium	525 mg	8.4%	9.7%	3.1%	1.7%	5.1%	1.4%	3.0%	1.4%	5.6%	2.8%
Magnesium	M: 250 mg, W: 200 mg	6.4%	12.6%	1.3%	4.0%	1.0%	1.4%	1.7%	3.7%	1.5%	2.5%
Iron *	M, and W aged 50+: 6.7 mg	0.8%	1.9%	0.4%	0.0%	0.6%	1.4%	0.6%	0.9%	0.2%	0.3%
Bioavailability adjusted Iron *	M, and W aged 50+: 12.06 mg ^c^							16.0%	19.4%	6.7%	9.3%
Zinc	M: 7.3 mg, W: 5.5 mg	0.9%	5.2%	2.0%	7.4%	4.7%	4.2%	4.2%	10.6%	6.3%	13.3%
Bioavailability adjusted Zinc	M: 10.95 mg, W: 8.25 mg ^c^							31.5%	47.2%	34.8%	49.9%
Iodine	95 µg ^c^	1.9%	1.0%	5.1%	5.1%	5.9%	8.5%	26.3%	16.7%	67.3%	60.9%
Selenium	45 µg ^d^	6.4%	6.8%	11.4%	10.8%	17.4%	11.3%	67.3%	50.0%	68.0%	54.7%

EAR—estimated average requirement; M—men; W—women; REs—retinol equivalents. ^a^ Source: unless specified otherwise, Department of Health, 1991. Dietary reference values for food, energy, and nutrients in the United Kingdom. ^b^ Source: Institute of Medicine, 2005. Dietary Reference Intakes for Energy, Carbohydrate, Fiber, Fat, Fatty Acids, Cholesterol, Protein, and Amino Acids. ^c^ Source: Institute of Medicine, 2001. Dietary reference intakes for vitamin A, vitamin K, arsenic, boron, chromium, copper, iodine, iron, manganese, molybdenum, nickel, silicon, vanadium, and zinc. Bioavailability adjustment for iron: EAR plus 80% for vegetarians and vegans; bioavailability adjustment for zinc: EAR plus 50% for vegetarians and vegans. ^d^ Source: Institute of Medicine, 2000. Dietary reference intakes for vitamin C, vitamin E, selenium, and carotenoids. Exclusions: * Younger women (<50 years), 100 participants who preferred not to state their gender.

## Data Availability

The data access policy for the FEED study is available via the study website: https://www.ceu.ox.ac.uk/research/feeding-the-future-study-feed.

## Supplementary Materials

The following supporting information can be downloaded at: https://www.mdpi.com/article/10.3390/nu16091336/s1, Table S1. Food items within each major food group. Table S2. (a) Mean intakes of major food groups in women, standardised to a 2000 kcal daily diet, (b) mean intakes of major food groups in men, standardised to a 2000 kcal daily diet. Table S3. Absolute mean intakes of major food groups (combined). Table S4. (a) Mean daily dietary nutrient intakes for women (unadjusted), (b) mean daily dietary nutrient intakes for men (unadjusted). Table S5. (a) Supplement use in women, by diet group, (b) supplement use in men, by diet group. Table S6. Mean daily dietary nutrient intakes: results from sensitivity analysis (includes vitamins, minerals, and supplements).

## Appendix B

### Appendix B.1. Additional Foods

The plant-based milk alternatives and other meat alternatives chosen as representative for nutritional information were selected based on unique products (combination of brand and specific product type) that were reported at least 10 times in the free text. For milk alternatives, the selection was assigned to participants by the type of milk specified (soya, rice, oat, pea, almond, coconut, hemp, other). For protein shakes, bars, and meal replacements, the selection was based on brands that were reported at least 10 times, with specific products selected based on what is available from those brands (e.g., all whey protein products available from the brands reported at least 10 times). Nutrients from protein shakes and meal replacement products were assigned to participants based on the type of protein source specified (whey, rice, soya, pea, hemp, peanut, other). Protein shake brands containing a blend of protein sources were represented in the “other” category. The same process was repeated for protein bar brands; however, as these usually contain a blend of protein sources, the selection for each protein source was based on the predominant ingredient (determined by % or order of ingredients list).

### Appendix B.2. Missing Values

Information from online retailers was generally limited to mandatory reporting regulations (energy value, total fat, saturated fat, carbohydrates, sugars, protein, salt) and occasionally additional information, for example, if the product is fortified. Estimates were extrapolated from the corresponding foods in the NDB to complete missing nutrient values where possible. In instances where a corresponding food was not available, a similar food was used, e.g., soya beans for tempeh. If several food codes were available in the NDB for a particular survey item, the mean was used to interpolate values. In instances where the product could be either fortified or non-fortified (e.g., plant milk), the selection of food codes used to calculate the average was additionally restricted to include only corresponding NDB entries. The NDB did not contain any similar foods for one product (seitan), so an alternative European food composition table, the FRIDA Danish food composition database, was used [68].

### Appendix B.3. Mapping

Each survey item was mapped to food codes in the database based on the wording of the questionnaire (foods specifically asked within the question) and any additional codes perceived to be common foods in the British diet (based on the NDNS and discussion in the research group). The selected food codes were cross-checked for consistency with EPIC-Oxford publications and the UKB Oxford WebQ nutrient update [19]. The selection of food codes for specific survey items was adapted where participants were able to specify consuming fortified or non-fortified versions of a food.

For most of the FFQ items, non-fortified food codes were selected unless otherwise specified or assumed. For foods where fortification was explicitly asked (plant-based dairy alternatives and plant-based meat alternatives), weighting was adjusted accordingly to include only codes with the corresponding fortification specifications. If participants did not specify or selected “don’t know”, all available codes for the item were averaged. For survey items where a distinction was made between dairy and non-dairy, a subset of plant-based codes was selected that was assigned to vegans only. A total of 430 foods codes from the NDB were mapped to survey items. An additional 145 foods were incorporated from FoodDB/online retailers.
