# Correction: Lawson et al. Assessing Performance of Contemporary Plant-Based Diets Against the UK Dietary Guidelines: Findings from the Feeding the Future (FEED) Study. *Nutrients* 2024, *16*, 1336

**DOI:** 10.3390/nu18193109

**Published:** 2026-09-22

**Authors:** Izabella Lawson, Caroline Wood, Nandana Syam, Holly Rippin, Selina Dagless, Kremlin Wickramasinghe, Birdem Amoutzopoulos, Toni Steer, Timothy J. Key, Keren Papier

**Affiliations:** 1 Cancer Epidemiology Unit, Nuffield Department of Population Health, University of Oxford, Oxford OX3 7LF, UK; izabella.lawson@ndph.ox.ac.uk (I.L.); tim.key@ceu.ox.ac.uk (T.J.K.); 2 Public Affairs & Communications Directorate, University of Oxford, Oxford OX1 2JD, UK; 3 Medical Sciences Division, University of Oxford, Oxford OX3 9DU, UK; 4 Special Initiative on NCDs and Innovation, World Health Organization Regional Office for Europe, DK-2100 Copenhagen, Denmark; 5 MRC Epidemiology Unit, University of Cambridge School of Clinical Medicine, Cambridge Biomedical Campus, Cambridge CB2 0SL, UK

The original publication [1] contained an error in the data processing for body weight, which affected the calculation of body mass index (BMI) and basal metabolic rate (BMR). Consequently, Figure 1 and several table values were incorrect (in Tables 1, 3, 4, S4a,b and S6). The corrected text, tables, and figure are below. And the corrected Supplementary Tables S4 and S6 attached. The authors state that the scientific conclusions are unaffected. This correction was approved by the Academic Editor. The original publication has also been updated.

## Text Corrections

A correction has been made to the affected text in the Results Section:

Large differences in BMI were observed across diet groups; BMI was highest in omnivores (26.2 kg/m^2^), followed by vegetarians (24.9 kg/m^2^) and pescatarians (24.4 kg/m^2^), and lowest in the flexitarians and vegans (24.2 kg/m^2^ and 24.3 kg/m^2^, respectively). Omnivores had the highest proportion of obese adults of all diet groups (16.8%). And the sentence “Participants were on average overweight (mean BMI 28.0 kg/m^2^), with 32.5% classified as obese.” was deleted.

Table 4 shows the proportion of women and men in each diet group who did not meet the recommended dietary targets. Some inadequacy for protein was observed in vegetarians and vegans (~10–15%).

A correction has been made to the affected text in the Discussion Section:

Among FEED participants, protein intake was fairly high on average, with some inadequacy observed across the diet groups (<5% of omnivores and flexitarians, and 10–15% of vegans and vegetarians not meeting the EAR).

## Table Corrections

**Table 1:** Baseline characteristics of the FEED cohort by diet group.

	Omnivorous	Flexitarian	Pescatarian	Vegetarian	Vegan	All	*p*-Value
*N*	1562 (24.6%)	1349 (21.3%)	568 (9.0%)	1292 (20.4%)	1571 (24.8%)	6342 (100.0%)	
Length following diet							
<1 year	56 (3.6%)	90 (6.7%)	23 (4.0%)	23 (1.8%)	106 (6.7%)	298 (4.7%)	<0.001
1–4 years	161 (10.3%)	386 (28.6%)	138 (24.3%)	148 (11.5%)	572 (36.4%)	1405 (22.2%)	
5–19 years	154 (9.9%)	463 (34.3%)	154 (27.1%)	212 (16.4%)	648 (41.2%)	1631 (25.7%)	
20+ years	1191 (76.2%)	410 (30.4%)	253 (44.5%)	909 (70.4%)	245 (15.6%)	3008 (47.4%)	
Age (years)	52.8 ± 14.6	52.0 ± 16.9	53.3 ± 16.3	53.0 ± 16.2	48.1 ± 15.2	51.5 ± 15.9	<0.001
Gender							
Female	1243 (79.6%)	1153 (85.5%)	493 (86.8%)	1060 (82.0%)	1167 (74.3%)	5116 (80.7%)	
Male	310 (19.8%)	176 (13.0%)	71 (12.5%)	216 (16.7%)	353 (22.5%)	1126 (17.8%)	<0.001
Prefer not to say	9 (0.6%)	20 (1.5%)	4 (0.7%)	16 (1.2%)	51 (3.2%)	100 (1.6%)	
BMI (kg/m^2^)	26.2 ± 5.8	24.2 ± 4.9	24.4 ± 5.0	24.9 ± 5.4	24.3 ± 4.9	24.9 ± 5.3	<0.001
Ethnicity							
White	1468 (94.0%)	1254 (93.0%)	536 (94.4%)	1245 (96.4%)	1482 (94.3%)	5985 (94.4%)	0.012
Black	4 (0.3%)	10 (0.7%)	5 (0.9%)	3 (0.2%)	4 (0.3%)	26 (0.4%)	
Asian	39 (2.5%)	47 (3.5%)	12 (2.1%)	23 (1.8%)	32 (2.0%)	153 (2.4%)	
Other	28 (1.8%)	23 (1.7%)	11 (1.9%)	11 (0.9%)	30 (1.9%)	103 (1.6%)	
Do not want to disclose	23 (1.5%)	15 (1.1%)	4 (0.7%)	10 (0.8%)	23 (1.5%)	75 (1.2%)	
Overall health							
Good or excellent	1204 (77.1%)	1077 (79.8%)	456 (80.3%)	969 (75.0%)	1293 (82.3%)	4999 (78.8%)	<0.001
Fair	308 (19.7%)	232 (17.2%)	98 (17.3%)	258 (20.0%)	217 (13.8%)	1113 (17.5%)	
Poor	47 (3.0%)	38 (2.8%)	14 (2.5%)	59 (4.6%)	54 (3.4%)	212 (3.3%)	
Do not know/prefer not to say	3 (0.2%)	2 (0.1%)	0 (0.0%)	6 (0.5%)	7 (0.4%)	18 (0.3%)	
Ever smoked tobacco							
No, never	958 (61.3%)	886 (65.7%)	341 (60.0%)	827 (64.0%)	944 (60.1%)	3956 (62.4%)	0.004
Yes, in the past	524 (33.5%)	422 (31.3%)	202 (35.6%)	428 (33.1%)	558 (35.5%)	2134 (33.6%)	
Yes, now	80 (5.1%)	41 (3.0%)	25 (4.4%)	37 (2.9%)	69 (4.4%)	252 (4.0%)	
Vigorous exercise in the last 12 months							
No	340 (21.8%)	266 (19.7%)	115 (20.2%)	327 (25.3%)	301 (19.2%)	1349 (21.3%)	<0.001
Yes	1222 (78.2%)	1083 (80.3%)	453 (79.8%)	965 (74.7%)	1270 (80.8%)	4993 (78.7%)	
Highest attained education							
GCSEs *	127 (8.1%)	71 (5.3%)	37 (6.5%)	101 (7.8%)	121 (7.7%)	457 (7.2%)	<0.001
A levels ^Ɨ^	225 (14.4%)	155 (11.5%)	73 (12.9%)	221 (17.1%)	273 (17.4%)	947 (14.9%)	
University degree	1163 (74.5%)	1101 (81.6%)	441 (77.6%)	934 (72.3%)	1128 (71.8%)	4767 (75.2%)	
None	12 (0.8%)	7 (0.5%)	7 (1.2%)	16 (1.2%)	17 (1.1%)	59 (0.9%)	
Do not want to disclose	35 (2.2%)	15 (1.1%)	10 (1.8%)	20 (1.5%)	32 (2.0%)	112 (1.8%)	
Paid job at present							
Yes	961 (61.5%)	792 (58.7%)	332 (58.5%)	729 (56.4%)	1020 (64.9%)	3834 (60.5%)	<0.001
No	131 (8.4%)	108 (8.0%)	48 (8.5%)	127 (9.8%)	197 (12.5%)	611 (9.6%)	
Retired	429 (27.5%)	424 (31.4%)	178 (31.3%)	405 (31.3%)	300 (19.1%)	1736 (27.4%)	
Do not want to disclose	41 (2.6%)	25 (1.9%)	10 (1.8%)	31 (2.4%)	54 (3.4%)	161 (2.5%)	
Average total income before tax received by household							
<GBP 18,000-GBP 49,999	673 (43.1%)	624 (46.3%)	258 (45.4%)	586 (45.4%)	808 (51.4%)	2949 (46.5%)	<0.001
GBP 50,000–GBP 100,000	585 (37.5%)	474 (35.1%)	197 (34.7%)	396 (30.7%)	460 (29.3%)	2112 (33.3%)	
Do not want to disclose	304 (19.5%)	251 (18.6%)	113 (19.9%)	310 (24.0%)	303 (19.3%)	1281 (20.2%)	
Region							
England							
East Midlands	205 (13.2%)	130 (9.7%)	73 (12.9%)	161 (12.5%)	210 (13.4%)	779 (12.3%)	<0.001
Greater London	131 (8.4%)	207 (15.5%)	81 (14.3%)	157 (12.2%)	191 (12.2%)	767 (12.2%)	
North East	82 (5.3%)	55 (4.1%)	39 (6.9%)	88 (6.8%)	109 (7.0%)	373 (5.9%)	
North West	109 (7.0%)	92 (6.9%)	38 (6.7%)	131 (10.2%)	169 (10.8%)	539 (8.5%)	
South East	542 (34.9%)	442 (33.0%)	163 (28.8%)	320 (24.8%)	349 (22.3%)	1816 (28.8%)	
South West	189 (12.2%)	180 (13.5%)	78 (13.8%)	150 (11.6%)	217 (13.9%)	814 (12.9%)	
West Midlands	125 (8.0%)	94 (7.0%)	36 (6.4%)	117 (9.1%)	112 (7.2%)	484 (7.7%)	
Northern Ireland	12 (0.8%)	7 (0.5%)	3 (0.5%)	13 (1.0%)	19 (1.2%)	54 (0.9%)	
Scotland	93 (6.0%)	85 (6.4%)	29 (5.1%)	100 (7.8%)	95 (6.1%)	402 (6.4%)	
Wales	66 (4.2%)	46 (3.4%)	26 (4.6%)	52 (4.0%)	91 (5.8%)	281 (4.5%)	

Data are presented as mean ± SD for continuous measures, and *n* (%) for categorical measures. *p*-Values for ANOVA and Chi2 tests. * GCSE—qualification in a specific subject typically taken by school students aged 14–16 in the UK except Scotland. ^Ɨ^ A-Levels (Advanced Level qualifications)—UK subject-based qualification for students aged 16 and above (includes Scottish Highers).

**Table 3:** Daily dietary nutrient intakes, adjusted for age and gender.

	Omnivores	Flexitarians	Pescatarians	Vegetarians	Vegans
Energy (kJ)	9511 ^b^	9388 ^b^	9277 ^a,b^	9047 ^a^	9132 ^a^
Energy/BMR ratio	1.59 ^a,b^	1.63 ^b^	1.60 ^a,b^	1.55 ^a^	1.59 ^a^
Energy/BMR ratio < 1.2 (%)	18.8 ^a,b^	17.2 ^a^	19.1 ^a,b^	21.9 ^b^	21.1 ^a,b^
Carbohydrate (%E)	37.8	44.8	46.4	48.9 ^a^	49.2 ^a^
Total sugars (%E)	17.4	19.9 ^a^	20.1 ^a,b^	20.6 ^b^	20.2 ^a,b^
Free sugars (%E)	7.67 ^a,b^	7.85 ^b^	8.02 ^b^	8.99	7.40 ^a^
Starch (%E)	20.5	25.0	26.4	28.4	30.8
Protein (%E)	17.8	15.1	14.2	12.6	12.2
Protein (g) per kg body weight	1.49	1.34	1.24	1.06 ^a^	1.06 ^a^
Fat (%E)	40.4	36.2 ^b^	35.3 ^a,b^	34.8 ^a^	35.1 ^a^
SFA (%E)	14.2	10.9	9.9 ^a^	10.2 ^a^	7.9
MUFA (%E)	16.0	14.9 ^b^	14.7 ^a,b^	14.1 ^a^	14.9 ^b^
PUFA (%E)	6.5	7.0	7.4 ^a^	7.4 ^a^	9.2
P/S ratio	0.50	0.68	0.78 ^a^	0.76 ^a^	1.21
Cholesterol (mg)	415	248	217	168	20
*n*-3 fatty acids (g)	2.83 ^a^	2.80 ^a^	2.86 ^a^	2.35	2.83 ^a^
*n*-6 fatty acids (g)	13.8	15.1 ^a^	15.7 ^a,b^	15.9 ^b^	20.2
Trans fatty acids (g)	1.45	0.95 ^a^	0.77 ^a^	0.80	0.38
Alcohol (%E)	2.85 ^a^	2.85 ^a^	3.16 ^a^	2.72 ^a^	2.23
Alcohol (g)	9.38 ^a,b^	9.24 ^a,b^	10.26 ^b^	8.45 ^a^	6.98
AOAC Fibre (g)	28.4	36.7	39.3 ^a^	38.8 ^a^	46.7
β-Carotene (μg)	3668	4240 ^a^	4204 ^a^	4039 ^a^	4859
Retinol (μg)	997	466	272 ^a^	259 ^a^	102
Vitamin A (RE) (µg)	1682	1261	1057 ^a^	1009 ^a^	989 ^a^
Vitamin D (μg)	5.57	4.82 ^a^	4.82 ^a^	3.07	2.60
Thiamin (mg)	2.07	2.19 ^a^	2.23 ^a,b^	2.28 ^b^	2.62
Riboflavin (mg)	2.26	2.03 ^b^	1.96 ^a,b^	1.94 ^a^	1.80
Niacin equivalent (mg)	47.0	40.2	36.9	31.9	33.3
Vitamin C (mg)	151	170 ^a^	175 ^a^	167 ^a^	193
Vitamin E (mg)	14.1	15.7 ^a^	16.7 ^b^	16.0 ^a,b^	18.7
Vitamin B6 (mg)	2.30	2.15	2.05 ^a^	1.90	2.04 ^a^
Vitamin B12 (μg)	9.78	6.49	5.55	3.35	2.04
Folate (μg)	362	401 ^a^	413 ^a^	411 ^a^	453
Pantothenic acid (mg)	8.25	7.42	6.90	6.42	5.84
Biotin (µg)	56.2	59.4 ^a^	61.4 ^a^	59.2 ^a^	67.4
Sodium (mg)	1973	1816 ^b^	1769 ^a,b^	1761 ^a,b^	1712 ^a^
Potassium (mg)	3923 ^a^	4072 ^b^	4085 ^b^	3836 ^a^	4144 ^b^
Calcium (mg)	976	1031 ^a^	1054 ^a^	1055 ^a^	1022 ^a^
Magnesium (mg)	363	414 ^a^	430 ^a^	413 ^a^	478
Phosphorus (mg)	1619	1557 ^b^	1537 ^b^	1420 ^a^	1397 ^a^
Iron (mg)	13.9	15.1 ^a^	15.4 ^a^	15.2 ^a^	17.6
Haem iron (mg)	1.05	0.49	0.30	0.16	0.18 ^a^
Non-haem iron (mg)	12.8	14.5 ^a^	15.1 ^a,b^	15.1 ^b^	17.3
Copper (mg)	1.80 ^a^	1.86 ^a^	1.86 ^a^	1.81 ^a^	2.20
Zinc (mg)	12.4	10.8	10.3 ^a^	10.1 ^a^	10.0 ^a^
Chloride (mg)	3472	3269 ^a,b^	3177 ^a^	3166 ^a^	3363 ^b^
Iodine (μg)	232	203 ^a^	200 ^a^	135	89
Manganese (mg)	4.23	5.64	6.12 ^a^	6.14 ^a^	7.37
Selenium (μg)	79.7	67.5 ^b^	66.4 ^b^	43.2 ^a^	41.3 ^a^

Energy/BMR ratio—energy intake to basal metabolic rate ratio; SFAs—saturated fatty acids; PUFAs—polyunsaturated fatty acids; P/S ratio—polyunsaturated fat (g)/saturated fat (g); values are presented as means adjusted for age and gender. ANOVA was used to compare the means between the diet groups. Multiple pairwise comparisons with Bonferroni correction were used to determine the statistical significance of differences in intakes between pairs of diet groups. ^a,b^ Pairs of means in the same row sharing a common superscript are not significantly different at the 5% level. *p*-Values for heterogeneity between diet groups for all nutrients were less than 0.0001. *N* = 6342: 1562 omnivores, 1349 flexitarians, 568 pescatarians, 1292 vegetarians, and 1571 vegans.

**Table 4:** Prevalence of inadequate intakes by gender and diet group.

Nutrient	EAR Value ^a^	Omnivores		Flexitarians		Pescatarians		Vegetarians		Vegans	
		W	M	W	M	W	M	W	M	W	M
Protein	0.66 g/kg of body weight ^b^	1.6%	3.3%	3.1%	5.8%	5.5%	5.8%	11.6%	10.7%	13.7%	15.0%
Vitamin A (RE)	M: 500 µg, W: 400 µg	1.2%	4.2%	1.5%	5.1%	2.6%	8.5%	4.8%	8.3%	8.9%	19.3%
Thiamin	0.3 mg per 1000 kcal energy	0.0%	0.0%	0.0%	0.0%	0.0%	0.0%	0.0%	0.0%	0.0%	0.0%
Riboflavin	M: 1.0 mg, W: 0.9 mg	0.6%	1.9%	1.7%	2.8%	3.4%	1.4%	2.7%	1.4%	5.6%	7.4%
Niacin	5.5 mg per 1000 kcal energy	0.0%	0.0%	0.0%	0.0%	0.0%	0.0%	0.0%	0.0%	0.0%	0.0%
Vitamin B6	13 µg per g protein	0.9%	1.3%	0.3%	0.0%	0.0%	0.0%	0.0%	0.0%	0.3%	0.3%
Vitamin B12	1.25 µg	0.1%	0.0%	0.8%	0.0%	0.2%	1.4%	4.7%	3.2%	31.2%	30.0%
Folate	150 µg	3.7%	2.9%	0.6%	0.0%	0.6%	0.0%	0.8%	0.0%	0.8%	0.0%
Vitamin C	25 mg	4.1%	6.1%	0.2%	0.0%	0.2%	0.0%	0.0%	0.0%	0.1%	0.0%
Calcium	525 mg	8.4%	9.7%	3.1%	1.7%	5.1%	1.4%	3.0%	1.4%	5.6%	2.8%
Magnesium	M: 250 mg, W: 200 mg	6.4%	12.6%	1.3%	4.0%	1.0%	1.4%	1.7%	3.7%	1.5%	2.5%
Iron *	M, and W aged 50+: 6.7 mg	0.8%	1.9%	0.4%	0.0%	0.6%	1.4%	0.6%	0.9%	0.2%	0.3%
Bioavailability adjusted Iron *	M, and W aged 50+: 12.06 mg ^c^							16.0%	19.4%	6.7%	9.3%
Zinc	M: 7.3 mg, W: 5.5 mg	0.9%	5.2%	2.0%	7.4%	4.7%	4.2%	4.2%	10.6%	6.3%	13.3%
Bioavailability adjusted Zinc	M: 10.95 mg, W: 8.25 mg ^c^							31.5%	47.2%	34.8%	49.9%
Iodine	95 µg ^c^	1.9%	1.0%	5.1%	5.1%	5.9%	8.5%	26.3%	16.7%	67.3%	60.9%
Selenium	45 µg ^d^	6.4%	6.8%	11.4%	10.8%	17.4%	11.3%	67.3%	50.0%	68.0%	54.7%

EAR—estimated average requirement; M—men; W—women; REs—retinol equivalents. ^a^ Source: unless specified otherwise, Department of Health, 1991. Dietary reference values for food, energy, and nutrients in the United Kingdom. ^b^ Source: Institute of Medicine, 2005. Dietary Reference Intakes for Energy, Carbohydrate, Fiber, Fat, Fatty Acids, Cholesterol, Protein, and Amino Acids. ^c^ Source: Institute of Medicine, 2001. Dietary reference intakes for vitamin A, vitamin K, arsenic, boron, chromium, copper, iodine, iron, manganese, molybdenum, nickel, silicon, vanadium, and zinc. Bioavailability adjustment for iron: EAR plus 80% for vegetarians and vegans; bioavailability adjustment for zinc: EAR plus 50% for vegetarians and vegans. ^d^ Source: Institute of Medicine, 2000. Dietary reference intakes for vitamin C, vitamin E, selenium, and carotenoids. Exclusions: * Younger women (<50 years), 100 participants who preferred not to state their gender.

## Figure Corrections

A correction has been made to Figure 1.

**Figure 1:**
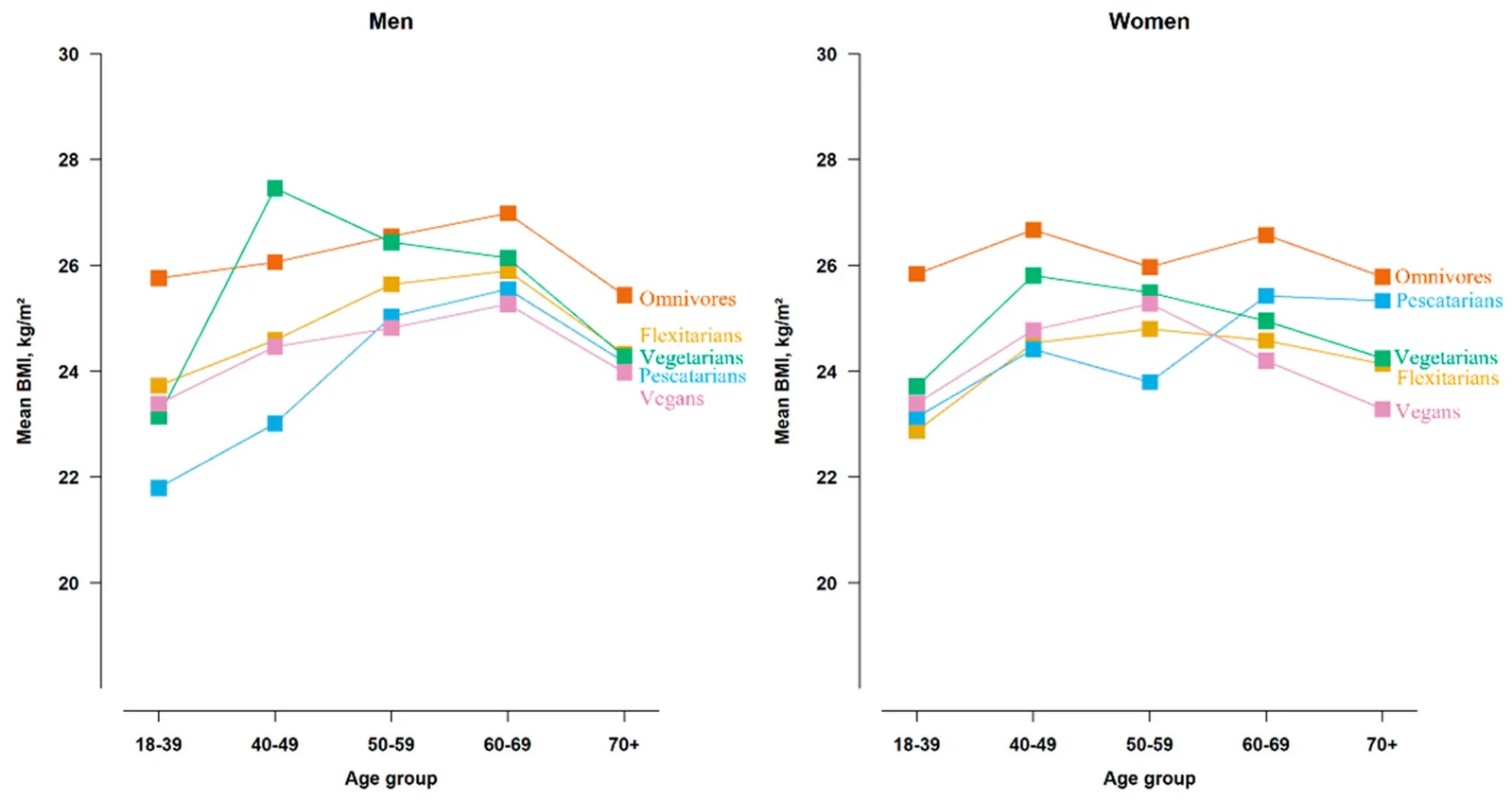
Mean body mass index (BMI) by age and diet group. Exclusions: 319 participants with missing values for height and weight, 100 participants who preferred not to state their gender.
