# Supplementary material for: Assessing Performance of Contemporary Plant-Based Diets against the UK Dietary Guidelines: Findings from the Feeding the Future (FEED) Study
Source: Nutrients. 2024 Apr 29;16(9):1336. doi: 10.3390/nu16091336 (PMC11085280; doi:10.3390/nu16091336)
Supplement: Supplementary file 1 [file nutrients-16-01336-s001.zip › nutrients-2965352-supplementary.pdf]

Table S1. Food items within each major food group.

Food Group	Food Items
Meat	Beef, pork, lamb, chicken/turkey, offal, sausages , bacon, salami/dried meats, sandwich slices, other processed meat (e.g. nuggets, burgers, pies, sausage roll)
Fish	Tuna, fish fried in batter/crumbs, white fish, oily fish, shellfish
Plant-based meat alternatives	Tofu, tempeh, textured vegetable protein, Quorn, falafel, soya burgers or sausages, other veggie burgers or sausages, seitan, other meat alternatives
Eggs	Eggs
Milk	Full cream cows' milk, semi-skimmed cows' milk, skimmed/fat free cows' milk, goats' milk, lactose free milk
Plant-based milk alternatives	Soya milk, rice milk, oat milk, pea milk, almond milk, coconut milk, hemp milk, other milk alternatives (hazlenut, cashew)
Cheese	Dairy cheese
Plant-based cheese alternatives	Vegan cheese
Yogurt	Dairy low fat yogurt, dairy full fat yogurt
Plant-based yogurt alternatives	Vegan yogurt
Pulses	Hummus, chickpeas, baked beans, other beans or lentils
Nuts/seeds	Peanut/other nut butters (with added sugar), peanut/other nut butters (without sugar), tahini, peanuts/other nuts or seeds (unsalted), peanuts/other nuts or seeds (salted)
Grains	White bread, wholemeal bread, brown/ granary bread, other bread products, oatcakes, crackers, crispbread, porridge/Ready brek/overnight oats, All-Bran, bran flakes/Weetabix/muesli, sugary cereal, other breakfast cereal, white rice, brown rice, couscous/bulgur wheat, white pasta/noodles, wholemeal pasta/noodles, pizza
Vegetables	Tomatoes, salad vegetables, carrots, parsnips/turnips/swedes/beetroot/other root vegetables, peas/green beans, broccoli/spring greens/kale/spinach, cabbage/Brussels sprouts/cauliflower/coleslaw, onions/leeks, mushrooms, sweetcorn, avocado, sweet pepper, other vegetables (e.g. courgette, pumpkin)
Fruit	Apples/pears, oranges/satsumas/grapefruit, banana, other fresh fruit (e.g. grapes, berries, kiwi), tinned or stewed fruit, dried fruit
Confectionery	Biscuits, cakes/pastries, cereal bar, nut and/or dried fruit bar, fruit pie/fruit tart/crumble, rice pudding/custard/crème caramel/mousse, vegan ice cream/choc ices, dairy ice cream/choc ices, chocolate/sweets, added sugar
Protein shakes/bars	Protein shake, protein bar
Meal replacements	Huel shake/bar , other meal replacement shake/bar
Tea/coffee	Tea, coffee (all types)
Non-alcoholic drinks	Hot chocolate/milkshake (incl. non-dairy), milk-based smoothie (incl. non-dairy), pure fruit juice/smoothie, fruit squash/cordial, low calorie soft drinks /diet fizzy soft drinks, fizzy soft drinks
Alcoholic drinks	Wine/champagne/sherry, beer/lager/cider, spirits

Table S2a. Mean intakes of major food groups in women, standardised to a 2000 kcal daily diet.

	Omnivorous	Flexitarian	Pescatarian	Vegetarian	Vegan	*p*-Value
N	1,243 (24.3%)	1,153 (22.5%)	493 (9.6%)	1,060 (20.7%)	1,167 (22.8%)	
Meat (g/2000 kcal)	122.5 ± 99.3	43.5 ± 27.7	1.2 ± 7.2	0.0 ± 0.7	0.1 ± 1.5	<0.001
Fish (g/2000 kcal)	47.5 ± 28.2	46.1 ± 30.3	51.0 ± 31.1	1.0 ± 4.8	0.4 ± 3.4	<0.001
Plant-based meat alternatives (g/2000 kcal)	9.8 ± 12.9	28.6 ± 27.4	47.7 ± 33.7	57.7 ± 38.3	70.6 ± 42.9	<0.001
Eggs (g/2000 kcal)	43.3 ± 50.8	31.0 ± 36.8	34.1 ± 46.6	32.5 ± 49.6	0.6 ± 5.7	<0.001
Milk (ml/2000 kcal)	171.1 ± 147.7	129.5 ± 132.9	109.3 ± 130.9	98.3 ± 133.4	0.7 ± 11.7	<0.001
Plant-based milk alternatives (ml/2000 kcal)	33.9 ± 83.4	80.3 ± 123.8	104.5 ± 126.7	118.0 ± 143.0	220.6 ± 165.9	<0.001
Cheese (g/2000 kcal)	18.9 ± 18.2	15.4 ± 12.4	15.4 ± 12.9	18.5 ± 16.6	0.1 ± 1.4	<0.001
Plant-based cheese alternatives (g/2000 kcal)	0.4 ± 2.6	0.9 ± 3.1	1.5 ± 3.5	2.4 ± 4.6	8.0 ± 8.9	<0.001
Yogurt (g/2000 kcal)	46.3 ± 48.3	51.2 ± 55.0	47.2 ± 57.7	41.0 ± 52.0	0.4 ± 5.4	<0.001
Plant-based yogurt alternatives (g/2000 kcal)	2.3 ± 12.2	7.1 ± 21.0	8.9 ± 24.8	13.6 ± 28.4	35.1 ± 44.5	<0.001
Pulses (g/2000 kcal)	32.5 ± 29.9	54.5 ± 38.2	65.8 ± 43.2	70.0 ± 42.3	100.0 ± 57.8	<0.001
Nuts/seeds (g/2000 kcal)	14.4 ± 17.6	19.9 ± 18.9	21.3 ± 19.7	21.1 ± 18.3	30.6 ± 24.5	<0.001
Grains (g/2000 kcal)	196.4 ± 116.1	260.2 ± 100.7	276.1 ± 103.3	277.1 ± 100.9	300.9 ± 110.1	<0.001
Vegetables (g/2000 kcal)	326.8 ± 192.6	382.6 ± 201.8	398.6 ± 201.5	374.0 ± 184.8	443.9 ± 229.4	<0.001
Fruit (g/2000 kcal)	168.4 ± 132.4	232.0 ± 155.0	225.6 ± 158.1	217.0 ± 151.3	244.8 ± 202.4	<0.001
Confectionery (g/2000 kcal)	52.3 ± 39.7	55.5 ± 34.6	55.5 ± 37.6	64.7 ± 35.6	49.2 ± 31.8	<0.001
Protein shakes/bars (g/2000 kcal)	1.4 ± 6.8	1.6 ± 7.1	1.4 ± 5.4	1.9 ± 8.5	3.4 ± 12.0	<0.001
Meal replacements (g/2000 kcal)	0.9 ± 8.5	1.2 ± 11.4	0.5 ± 4.8	1.3 ± 10.6	2.0 ± 13.4	0.064
Tea/coffee (ml/2000 kcal)	594.5 ± 349.9	606.0 ± 349.8	616.3 ± 345.6	596.2 ± 363.5	575.7 ± 361.2	0.175
Non-alcoholic drinks (ml/2000 kcal)	239.4 ± 344.5	179.2 ± 255.8	193.8 ± 279.5	242.1 ± 323.0	233.7 ± 322.7	<0.001
Alcoholic drinks (ml/2000 kcal)	84.2 ± 114.3	89.3 ± 102.4	97.1 ± 113.2	93.0 ± 116.2	76.9 ± 114.1	0.001

ANOVA was used to compare the means between the diet groups; 100 participants who preferred not to state their gender were excluded.

Table S2b. Mean intakes of major food groups in men, standardised to a 2000 kcal daily diet.

	Omnivorous	Flexitarian	Pescatarian	Vegetarian	Vegan	P-value
N	310 (27.5%)	176 (15.6%)	71 (6.3%)	216 (19.2%)	353 (31.3%)	
Meat (g/2000 kcal)	130.1 ± 99.2	41.9 ± 25.7	2.2 ± 10.4	0.4 ± 3.3	0.2 ± 2.4	<0.001
Fish (g/2000 kcal)	47.3 ± 54.5	41.2 ± 28.8	53.4 ± 39.2	0.7 ± 4.1	0.4 ± 3.3	<0.001
Plant-based meat alternatives (g/2000 kcal)	8.7 ± 14.7	30.5 ± 28.8	42.0 ± 28.3	59.4 ± 37.4	71.4 ± 44.8	<0.001
Eggs (g/2000 kcal)	49.3 ± 68.8	27.8 ± 35.5	33.0 ± 38.1	32.8 ± 48.9	1.1 ± 11.8	<0.001
Milk (ml/2000 kcal)	189.8 ± 160.8	129.1 ± 142.5	97.0 ± 120.7	114.6 ± 131.4	1.5 ± 17.0	<0.001
Plant-based milk alternatives (ml/2000 kcal)	18.7 ± 59.3	64.0 ± 112.3	96.1 ± 143.0	92.4 ± 124.7	197.9 ± 141.7	<0.001
Cheese (g/2000 kcal)	17.6 ± 15.7	14.8 ± 12.6	14.7 ± 14.2	17.5 ± 15.1	0.1 ± 1.0	<0.001
Plant-based cheese alternatives (g/2000 kcal)	0.2 ± 1.2	1.0 ± 3.5	1.3 ± 3.9	2.3 ± 4.5	7.0 ± 8.0	<0.001
Yogurt (g/2000 kcal)	35.1 ± 42.0	44.5 ± 51.2	38.9 ± 51.2	38.6 ± 43.0	0.5 ± 5.8	<0.001
Plant-based yogurt alternatives (g/2000 kcal)	1.1 ± 6.7	6.5 ± 20.0	7.8 ± 19.2	7.0 ± 15.4	27.1 ± 44.2	<0.001
Pulses (g/2000 kcal)	27.4 ± 25.4	49.0 ± 38.9	74.3 ± 67.2	63.6 ± 40.0	103.9 ± 72.3	<0.001
Nuts/seeds (g/2000 kcal)	12.0 ± 15.0	20.7 ± 20.9	23.3 ± 20.3	19.7 ± 17.9	32.2 ± 26.6	<0.001
Grains (g/2000 kcal)	200.6 ± 117.6	267.5 ± 100.3	287.9 ± 136.0	297.4 ± 100.3	315.2 ± 120.1	<0.001
Vegetables (g/2000 kcal)	243.8 ± 173.0	290.3 ± 131.2	333.1 ± 178.4	313.5 ± 145.2	364.7 ± 190.1	<0.001
Fruit (g/2000 kcal)	151.6 ± 148.7	221.6 ± 138.2	223.5 ± 136.3	206.3 ± 144.9	244.0 ± 174.0	<0.001
Confectionery (g/2000 kcal)	48.6 ± 38.7	51.1 ± 33.6	40.9 ± 26.7	56.4 ± 33.0	46.7 ± 39.0	0.007
Protein shakes/bars (g/2000 kcal)	2.0 ± 7.7	3.6 ± 13.4	3.2 ± 10.8	1.4 ± 5.1	3.8 ± 11.0	0.018
Meal replacements (g/2000 kcal)	1.5 ± 10.4	3.9 ± 30.8	1.1 ± 7.7	0.9 ± 7.2	5.9 ± 31.5	0.039
Tea/coffee (ml/2000 kcal)	486.8 ± 357.0	546.1 ± 304.3	553.7 ± 267.2	564.5 ± 306.9	447.1 ± 320.8	<0.001
Non-alcoholic drinks (ml/2000 kcal)	234.3 ± 332.4	155.8 ± 200.3	133.8 ± 165.5	227.6 ± 271.9	206.6 ± 302.1	0.007
Alcoholic drinks (ml/2000 kcal)	167.7 ± 227.6	161.1 ± 172.8	226.6 ± 294.0	156.0 ± 195.8	115.4 ± 178.5	<0.001

ANOVA was used to compare the means between the diet groups; 100 participants who preferred not to state their gender were excluded.

Table S3. Absolute mean intakes of major food groups (combined).

	Omnivorous	Flexitarian	Pescatarian	Vegetarian	Vegan	*p*-Value
N	1,562 (24.6%)	1,349 (21.3%)	568 (9.0%)	1,292 (20.4%)	1,571 (24.8%)	
Meat (g/day)	137.9 ± 117.9	46.9 ± 29.9	1.2 ± 7.3	0.1 ± 2.0	0.1 ± 1.5	<0.001
Fish (g/day)	52.1 ± 41.0	48.5 ± 32.0	54.8 ± 36.9	0.9 ± 4.6	0.4 ± 3.6	<0.001
Plant-based meat alternatives (g/day)	10.9 ± 15.5	31.6 ± 31.2	50.6 ± 37.5	61.7 ± 43.8	76.4 ± 50.2	<0.001
Eggs (g/day)	47.9 ± 55.5	32.6 ± 38.9	38.1 ± 53.9	34.2 ± 50.6	0.8 ± 8.6	<0.001
Milk (ml/day)	196.9 ± 167.2	142.9 ± 148.4	117.8 ± 144.1	103.5 ± 133.8	0.9 ± 14.9	<0.001
Plant-based milk alternatives (ml/day)	33.9 ± 87.2	82.6 ± 121.3	112.1 ± 142.5	119.5 ± 144.1	226.5 ± 161.7	<0.001
Cheese (g/day)	20.9 ± 20.7	17.0 ± 14.8	16.9 ± 15.8	19.6 ± 18.9	0.1 ± 1.4	<0.001
Plant-based cheese alternatives (g/day)	0.4 ± 3.2	1.0 ± 3.1	1.6 ± 4.2	2.5 ± 4.9	8.4 ± 9.6	<0.001
Yogurt (g/day)	49.5 ± 53.9	55.3 ± 61.9	49.0 ± 57.6	42.7 ± 52.0	0.4 ± 5.4	<0.001
Plant-based yogurt alternatives (g/day)	2.4 ± 14.3	7.6 ± 23.5	9.2 ± 24.4	13.8 ± 32.6	35.7 ± 48.0	<0.001
Pulses (g/day)	35.4 ± 31.3	58.6 ± 42.5	72.8 ± 57.8	72.6 ± 44.6	110.0 ± 78.3	<0.001
Nuts/seeds (g/day)	16.1 ± 21.2	22.9 ± 24.5	24.1 ± 24.3	23.5 ± 24.3	35.6 ± 33.6	<0.001
Grains (g/day)	228.9 ± 149.1	288.8 ± 129.1	302.8 ± 135.0	299.7 ± 128.4	331.4 ± 149.6	<0.001
Vegetables (g/day)	341.0 ± 207.3	395.4 ± 210.5	415.2 ± 213.8	381.6 ± 204.2	453.7 ± 243.7	<0.001
Fruit (g/day)	185.6 ± 154.1	249.5 ± 170.2	240.4 ± 164.8	223.5 ± 154.8	261.0 ± 203.7	<0.001
Confectionery (g/day)	60.2 ± 51.7	63.3 ± 49.0	59.6 ± 45.1	68.9 ± 46.2	53.7 ± 41.3	<0.001
Protein shakes/bars (g/day)	1.8 ± 8.3	2.2 ± 9.8	2.0 ± 8.6	1.9 ± 8.1	3.8 ± 12.8	<0.001
Meal replacements (g/day)	1.4 ± 11.3	1.9 ± 20.7	0.7 ± 6.0	1.5 ± 12.0	3.2 ± 20.9	0.005
Tea/coffee (ml/day)	618.4 ± 335.3	629.1 ± 318.0	628.9 ± 302.9	598.8 ± 320.3	566.4 ± 333.0	<0.001
Non-alcoholic drinks (ml/day)	268.4 ± 379.1	194.7 ± 266.7	199.8 ± 287.1	252.1 ± 322.8	234.3 ± 313.5	<0.001
Alcoholic drinks (ml/day)	115.7 ± 181.2	110.0 ± 140.6	127.4 ± 195.7	111.5 ± 155.6	93.9 ± 153.1	<0.001

ANOVA was used to compare the means between the diet groups.

Table S4a. Mean daily dietary nutrient intakes for women (unadjusted).

	Omnivorous	Flexitarian	Pescatarian	Vegetarian	Vegan
N	1,243 (24.3%)	1,153 (22.5%)	493 (9.6%)	1,060 (20.7%)	1,167 (22.8%)
Energy (kJ)	9305.1 ± 2256.3	9212.5 ± 2276.5	9013.4 ± 2296.3	8820.8 ± 2266.7	8871.1 ± 2382.2
Energy:BMR ratio	1.6 ± 0.4	1.7 ± 0.4	1.6 ± 0.4	1.6 ± 0.4	1.6 ± 0.5
Energy:BMR ratio <1.2 (%)	15.1	14.6	17.2	19.0	19.5
Carbohydrate (%E)	37.7 ± 13.8	44.6 ± 7.0	46.2 ± 7.2	48.6 ± 6.8	49.1 ± 6.8
Total sugars (%E)	17.5 ± 6.6	20.0 ± 5.2	20.3 ± 5.3	20.8 ± 5.1	20.2 ± 5.8
Free sugars (%E)	7.6 ± 4.5	7.8 ± 3.5	8.0 ± 3.8	8.9 ± 3.8	7.6 ± 3.8
Starch (%E)	20.3 ± 9.3	24.8 ± 6.1	26.1 ± 6.0	28.0 ± 5.9	30.7 ± 6.9
Protein (%E)	17.8 ± 4.4	15.1 ± 2.4	14.1 ± 2.3	12.5 ± 2.1	12.1 ± 2.4
Protein (g) per kg body weight	1.5 ± 0.6	1.4 ± 0.4	1.2 ± 0.4	1.1 ± 0.4	1.1 ± 0.4
Fat (%E)	40.8 ± 11.4	36.5 ± 6.5	35.7 ± 7.0	35.2 ± 6.3	35.4 ± 6.6
SFA (%E)	14.2 ± 5.5	11.0 ± 2.3	10.1 ± 2.3	10.3 ± 2.4	7.9 ± 1.7
MUFA (%E)	16.2 ± 5.0	15.0 ± 3.7	14.8 ± 4.0	14.2 ± 3.5	15.0 ± 3.8
PUFA (%E)	6.6 ± 1.7	7.1 ± 1.7	7.5 ± 1.9	7.4 ± 1.8	9.2 ± 2.0
PS ratio	0.5 ± 0.2	0.7 ± 0.2	0.8 ± 0.2	0.8 ± 0.2	1.2 ± 0.3
Cholesterol (mg)	403.0 ± 277.5	247.1 ± 159.3	211.8 ± 203.5	164.3 ± 177.5	18.8 ± 24.1
N-3 fatty acids (g)	2.8 ± 1.1	2.8 ± 0.9	2.8 ± 1.1	2.3 ± 0.9	2.8 ± 1.0
N-6 fatty acids (g)	13.7 ± 5.2	14.9 ± 5.5	15.5 ± 6.1	15.5 ± 6.2	19.6 ± 7.6
Trans fatty acids (g)	1.4 ± 0.9	0.9 ± 0.4	0.8 ± 0.4	0.8 ± 0.4	0.4 ± 0.2
Alcohol (%E)	2.6 ± 3.5	2.7 ± 3.3	3.0 ± 3.5	2.6 ± 3.2	2.1 ± 3.0
Alcohol (g)	8.5 ± 11.8	8.6 ± 10.8	9.1 ± 11.6	8.0 ± 10.0	6.4 ± 9.5
AOAC Fibre (g)	28.4 ± 12.1	36.4 ± 11.0	38.4 ± 11.8	38.1 ± 11.3	45.3 ± 14.6
β-Carotene (μg)	3788.5 ± 2353.6	4367.9 ± 2586.7	4228.4 ± 2290.7	4094.3 ± 2393.0	4824.5 ± 2850.9
Retinol (μg)	926.3 ± 1621.1	443.5 ± 375.1	259.5 ± 156.1	256.2 ± 175.7	88.3 ± 162.3
Vitamin A (retinol equivalents) (μg)	1637.3 ± 1629.6	1265.0 ± 633.7	1051.5 ± 462.0	1018.2 ± 485.7	978.8 ± 548.1
Vitamin D (μg)	5.5 ± 2.8	4.8 ± 2.4	4.7 ± 2.8	3.0 ± 1.9	2.5 ± 1.6
Thiamin (mg)	2.0 ± 0.6	2.2 ± 0.6	2.2 ± 0.6	2.2 ± 0.7	2.5 ± 0.8
Riboflavin (mg)	2.2 ± 0.7	2.0 ± 0.7	1.9 ± 0.7	1.9 ± 0.6	1.8 ± 0.7
Niacin equivalent (mg)	45.9 ± 13.7	39.5 ± 10.1	35.6 ± 9.8	30.8 ± 8.7	32.0 ± 9.9
Vitamin C (mg)	154.8 ± 79.1	172.8 ± 77.4	174.6 ± 75.9	167.1 ± 78.0	190.6 ± 99.5
Vitamin E (mg)	14.1 ± 5.3	15.5 ± 4.9	16.5 ± 5.6	15.8 ± 5.6	18.5 ± 6.3
Vitamin B6 (mg)	2.3 ± 0.6	2.1 ± 0.6	2.0 ± 0.6	1.8 ± 0.6	2.0 ± 0.7
Vitamin B12 (μg)	9.4 ± 6.7	6.4 ± 2.8	5.4 ± 2.6	3.3 ± 1.7	2.0 ± 1.3
Folate (μg)	359.5 ± 123.4	398.4 ± 121.4	403.4 ± 123.1	402.9 ± 129.0	437.5 ± 158.1
Pantothenic acid (mg)	8.1 ± 2.3	7.4 ± 2.1	6.8 ± 2.1	6.3 ± 2.0	5.6 ± 1.8
Biotin (μg)	55.1 ± 18.7	57.9 ± 19.3	59.4 ± 21.2	57.5 ± 20.5	65.0 ± 25.6
Sodium (mg)	1904.0 ± 567.6	1775.0 ± 535.7	1705.7 ± 538.9	1697.3 ± 545.1	1664.6 ± 583.4
Potassium (mg)	3895.2 ± 1059.5	4046.9 ± 1049.6	4009.2 ± 1051.5	3771.5 ± 1021.3	4026.9 ± 1243.3
Calcium (mg)	957.7 ± 322.8	1019.1 ± 322.1	1028.6 ± 332.8	1036.2 ± 309.8	1003.9 ± 346.4
Magnesium (mg)	358.4 ± 109.7	407.9 ± 109.0	418.4 ± 114.1	402.9 ± 115.0	461.7 ± 145.7
Phosphorus (mg)	1587.2 ± 399.9	1535.5 ± 390.9	1496.1 ± 399.4	1384.8 ± 373.7	1353.2 ± 399.5
Iron (mg)	13.6 ± 4.0	14.7 ± 4.0	14.9 ± 4.3	14.8 ± 4.3	17.0 ± 5.2
Haem iron (mg)	1.0 ± 0.9	0.5 ± 0.2	0.3 ± 0.2	0.1 ± 0.1	0.2 ± 0.2
Non-haem iron (mg)	12.5 ± 3.9	14.2 ± 3.9	14.6 ± 4.2	14.6 ± 4.2	16.7 ± 5.1
Copper (mg)	1.8 ± 0.9	1.8 ± 0.5	1.8 ± 0.6	1.8 ± 0.6	2.1 ± 0.7
Zinc (mg)	12.1 ± 4.3	10.6 ± 2.7	10.0 ± 2.8	9.8 ± 2.9	9.7 ± 3.0
Chloride (mg)	3365.4 ± 955.6	3202.1 ± 910.4	3070.3 ± 903.9	3060.4 ± 899.3	3276.3 ± 1132.2
Iodine (μg)	226.6 ± 75.8	201.8 ± 77.1	195.7 ± 79.3	132.4 ± 55.4	85.6 ± 35.6
Manganese (mg)	4.2 ± 1.9	5.5 ± 1.7	6.0 ± 1.8	6.0 ± 1.8	7.1 ± 2.2
Selenium (μg)	78.0 ± 27.2	67.0 ± 22.2	64.5 ± 24.3	41.9 ± 16.4	39.7 ± 14.0

Data are presented as mean ± SD.

Table S4b. Mean daily dietary nutrient intakes for men (unadjusted).

	**Omnivorous**	**Flexitarian**	**Pescatarian**	**Vegetarian**	**Vegan**
N	310 (27.5%)	176 (15.6%)	71 (6.3%)	216 (19.2%)	353 (31.3%)
Energy (kJ)	10366.1 ± 2791.2	10108.9 ± 2515.2	10622.0 ± 2776.7	10022.3 ± 2607.7	10183.1 ± 2697.7
Energy:BMR ratio	1.4 ± 0.4	1.5 ± 0.4	1.6 ± 0.4	1.5 ± 0.4	1.5 ± 0.4
Energy:BMR ratio <1.2 (%)	29.0	25.0	14.1	25.0	28.3
Carbohydrate (%E)	38.0 ± 14.5	45.2 ± 6.5	46.7 ± 7.3	50.1 ± 6.5	50.0 ± 7.1
Total sugars (%E)	16.9 ± 6.8	19.6 ± 5.2	19.9 ± 4.9	20.6 ± 6.0	19.7 ± 5.4
Free sugars (%E)	7.9 ± 4.4	8.0 ± 3.4	7.7 ± 3.0	9.1 ± 4.1	7.3 ± 3.9
Starch (%E)	21.1 ± 10.1	25.7 ± 5.9	26.9 ± 7.2	29.7 ± 5.7	31.8 ± 7.4
Protein (%E)	17.9 ± 5.2	14.9 ± 2.8	14.2 ± 2.5	12.7 ± 2.0	12.5 ± 2.3
Protein (g) per kg body weight	1.4 ± 0.6	1.2 ± 0.4	1.2 ± 0.4	1.0 ± 0.3	1.1 ± 0.4
Fat (%E)	39.0 ± 11.2	35.2 ± 6.1	33.4 ± 7.1	33.0 ± 5.9	33.8 ± 6.9
SFA (%E)	13.9 ± 5.5	10.5 ± 2.3	9.2 ± 2.3	9.5 ± 2.1	7.5 ± 1.8
MUFA (%E)	15.3 ± 4.6	14.5 ± 3.3	13.9 ± 3.8	13.5 ± 3.4	14.2 ± 3.9
PUFA (%E)	6.1 ± 1.5	6.9 ± 1.8	7.1 ± 1.9	7.1 ± 1.8	9.1 ± 2.2
PS ratio	0.5 ± 0.2	0.7 ± 0.3	0.8 ± 0.3	0.8 ± 0.3	1.3 ± 0.3
Cholesterol (mg)	468.8 ± 351.5	250.5 ± 161.0	232.8 ± 173.2	190.9 ± 228.2	21.8 ± 62.1
N-3 fatty acids (g)	2.9 ± 1.4	3.0 ± 1.3	3.2 ± 1.3	2.5 ± 1.1	3.0 ± 1.1
N-6 fatty acids (g)	14.1 ± 5.5	16.2 ± 6.7	16.9 ± 5.4	17.1 ± 7.3	22.3 ± 9.0
Trans fatty acids (g)	1.6 ± 0.9	1.0 ± 0.4	0.8 ± 0.5	0.8 ± 0.4	0.4 ± 0.3
Alcohol (%E)	4.0 ± 5.2	3.7 ± 3.6	4.7 ± 5.4	3.2 ± 3.9	2.4 ± 3.6
Alcohol (g)	14.0 ± 19.6	12.8 ± 12.8	18.1 ± 23.3	11.3 ± 15.1	8.5 ± 13.4
AOAC Fibre (g)	28.8 ± 13.6	38.2 ± 11.7	44.9 ± 14.1	42.1 ± 12.6	51.5 ± 16.7
β-Carotene (μg)	3237.0 ± 1979.9	3687.4 ± 1859.0	4437.9 ± 2263.9	3936.5 ± 2186.1	4655.0 ± 2626.6
Retinol (μg)	1286.6 ± 2579.6	551.6 ± 415.5	306.1 ± 203.8	276.9 ± 182.6	154.2 ± 451.8
Vitamin A (retinol equivalents) (μg)	1880.7 ± 2562.9	1238.3 ± 545.0	1143.7 ± 509.8	1010.8 ± 437.1	954.6 ± 507.1
Vitamin D (μg)	6.0 ± 4.1	5.0 ± 3.6	5.6 ± 3.6	3.3 ± 2.5	2.8 ± 2.1
Thiamin (mg)	2.2 ± 0.7	2.3 ± 0.7	2.5 ± 0.7	2.6 ± 0.9	2.9 ± 0.9
Riboflavin (mg)	2.5 ± 1.0	2.1 ± 0.7	2.2 ± 0.7	2.2 ± 0.7	1.9 ± 0.7
Niacin equivalent (mg)	51.7 ± 18.2	43.4 ± 11.7	43.9 ± 12.3	36.9 ± 10.1	38.1 ± 11.7
Vitamin C (mg)	139.3 ± 78.5	160.2 ± 70.9	186.8 ± 83.9	172.9 ± 85.3	192.5 ± 92.3
Vitamin E (mg)	14.0 ± 5.4	16.2 ± 5.7	17.9 ± 5.1	17.0 ± 6.5	19.4 ± 6.7
Vitamin B6 (mg)	2.5 ± 0.8	2.3 ± 0.6	2.4 ± 0.8	2.2 ± 0.7	2.3 ± 0.7
Vitamin B12 (μg)	11.6 ± 11.5	6.9 ± 3.5	6.4 ± 3.5	3.9 ± 1.9	2.2 ± 1.6
Folate (μg)	375.5 ± 131.2	417.9 ± 125.5	473.8 ± 136.2	456.4 ± 135.2	495.6 ± 167.7
Pantothenic acid (mg)	8.9 ± 2.8	7.7 ± 2.3	7.6 ± 2.6	7.2 ± 2.1	6.3 ± 2.0
Biotin (μg)	61.0 ± 21.0	65.9 ± 24.6	71.5 ± 21.0	67.1 ± 22.6	76.5 ± 30.5
Sodium (mg)	2256.1 ± 863.4	1973.8 ± 632.2	2066.9 ± 688.7	2032.2 ± 666.6	1924.4 ± 705.5
Potassium (mg)	4110.9 ± 1196.8	4224.9 ± 1010.5	4647.1 ± 1386.3	4241.9 ± 1146.2	4443.0 ± 1347.4
Calcium (mg)	1041.2 ± 400.3	1069.2 ± 356.5	1171.7 ± 394.9	1134.5 ± 336.3	1102.7 ± 380.9
Magnesium (mg)	384.3 ± 122.6	444.8 ± 125.5	496.3 ± 126.3	458.1 ± 129.9	534.1 ± 167.8
Phosphorus (mg)	1755.9 ± 515.5	1646.2 ± 435.3	1743.5 ± 505.2	1576.7 ± 409.7	1563.6 ± 460.3
Iron (mg)	15.1 ± 4.8	16.4 ± 4.6	18.1 ± 5.5	17.0 ± 5.1	20.1 ± 6.0
Haem iron (mg)	1.2 ± 1.3	0.5 ± 0.2	0.3 ± 0.2	0.2 ± 0.2	0.2 ± 0.2
Non-haem iron (mg)	13.8 ± 4.5	15.9 ± 4.6	17.8 ± 5.4	16.8 ± 5.0	19.8 ± 6.0
Copper (mg)	2.0 ± 1.4	2.0 ± 0.6	2.1 ± 0.6	2.0 ± 0.6	2.5 ± 0.8
Zinc (mg)	13.6 ± 5.6	11.5 ± 3.1	11.7 ± 3.4	11.2 ± 3.1	11.3 ± 3.5
Chloride (mg)	3922.4 ± 1370.3	3544.1 ± 1057.6	3716.8 ± 1230.1	3648.2 ± 1122.6	3721.2 ± 1304.2
Iodine (μg)	260.7 ± 122.8	208.8 ± 81.0	231.7 ± 109.2	152.1 ± 58.5	95.5 ± 41.1
Manganese (mg)	4.4 ± 2.2	6.1 ± 2.0	7.1 ± 2.0	6.9 ± 2.2	8.3 ± 2.6
Selenium (μg)	86.5 ± 43.4	69.7 ± 23.8	75.6 ± 30.4	48.9 ± 20.0	47.0 ± 17.2

Data are presented as mean ± SD.

Table S5a. Supplement use in women, by diet group.

	Omnivorous	Flexitarian	Pescatarian	Vegetarian	Vegan	*p*-Value
N	1,243 (24.3%)	1,153 (22.5%)	493 (9.6%)	1,060 (20.7%)	1,167 (22.8%)	
Take any vitamins, minerals, and/or supplements?						
Yes	843 (67.8%)	803 (69.6%)	355 (72.0%)	813 (76.7%)	1,027 (88.0%)	<0.001
No	392 (31.5%)	347 (30.1%)	137 (27.8%)	244 (23.0%)	138 (11.8%)	
prefer not to say	8 (0.6%)	3 (0.3%)	1 (0.2%)	3 (0.3%)	2 (0.2%)	
Multivitamins with minerals	260 (20.9%)	256 (22.2%)	154 (31.2%)	369 (34.8%)	552 (47.3%)	<0.001
Multivitamins without minerals	31 (2.5%)	29 (2.5%)	13 (2.6%)	45 (4.2%)	48 (4.1%)	0.027
Long-chain omega-3 fatty acids	194 (15.6%)	140 (12.1%)	65 (13.2%)	152 (14.3%)	279 (23.9%)	<0.001
Iron	100 (8.0%)	117 (10.1%)	73 (14.8%)	169 (15.9%)	200 (17.1%)	<0.001
Zinc	114 (9.2%)	76 (6.6%)	55 (11.2%)	77 (7.3%)	127 (10.9%)	<0.001
Calcium	115 (9.3%)	112 (9.7%)	58 (11.8%)	123 (11.6%)	141 (12.1%)	0.103
Iodine	32 (2.6%)	5 (0.4%)	8 (1.6%)	19 (1.8%)	99 (8.5%)	<0.001
Selenium	27 (2.2%)	12 (1.0%)	12 (2.4%)	30 (2.8%)	52 (4.5%)	<0.001
Vitamin A	14 (1.1%)	12 (1.0%)	13 (2.6%)	17 (1.6%)	18 (1.5%)	0.117
Folate	52 (4.2%)	38 (3.3%)	10 (2.0%)	29 (2.7%)	45 (3.9%)	0.119
Vit B12 tablets	97 (7.8%)	112 (9.7%)	78 (15.8%)	199 (18.8%)	441 (37.8%)	<0.001
Vit B12 spray	19 (1.5%)	14 (1.2%)	9 (1.8%)	20 (1.9%)	53 (4.5%)	<0.001
Vit B12 injections	17 (1.4%)	10 (0.9%)	5 (1.0%)	26 (2.5%)	22 (1.9%)	0.025
Vitamin C	136 (10.9%)	119 (10.3%)	60 (12.2%)	107 (10.1%)	111 (9.5%)	0.534
Vitamin D	502 (40.4%)	497 (43.1%)	204 (41.4%)	422 (39.8%)	456 (39.1%)	0.339
Vitamin E	21 (1.7%)	18 (1.6%)	17 (3.4%)	19 (1.8%)	23 (2.0%)	0.117
Superfoods	30 (2.4%)	49 (4.2%)	23 (4.7%)	36 (3.4%)	110 (9.4%)	<0.001
Other supplements	283 (22.8%)	203 (17.6%)	80 (16.2%)	187 (17.6%)	204 (17.5%)	0.001

Table S5b. Supplement use in men, by diet group.

	Omnivorous	Flexitarian	Pescatarian	Vegetarian	Vegan	*p*-Value
N	310 (27.5%)	176 (15.6%)	71 (6.3%)	216 (19.2%)	353 (31.3%)	
Take any vitamins, minerals, and/or supplements?						
Yes	169 (54.5%)	103 (58.5%)	51 (71.8%)	149 (69.0%)	302 (85.6%)	<0.001
No	139 (44.8%)	73 (41.5%)	20 (28.2%)	65 (30.1%)	50 (14.2%)	
prefer not to say	2 (0.6%)	0 (0.0%)	0 (0.0%)	2 (0.9%)	1 (0.3%)	
Multivitamins with minerals	70 (22.6%)	40 (22.7%)	18 (25.4%)	72 (33.3%)	188 (53.3%)	<0.001
Multivitamins without minerals	4 (1.3%)	4 (2.3%)	2 (2.8%)	7 (3.2%)	15 (4.2%)	0.234
Long-chain omega-3 fatty acids	37 (11.9%)	31 (17.6%)	10 (14.1%)	27 (12.5%)	92 (26.1%)	<0.001
Iron	9 (2.9%)	9 (5.1%)	4 (5.6%)	29 (13.4%)	31 (8.8%)	<0.001
Zinc	19 (6.1%)	24 (13.6%)	4 (5.6%)	14 (6.5%)	34 (9.6%)	0.030
Calcium	9 (2.9%)	7 (4.0%)	4 (5.6%)	8 (3.7%)	34 (9.6%)	0.001
Iodine	7 (2.3%)	3 (1.7%)	1 (1.4%)	2 (0.9%)	42 (11.9%)	<0.001
Selenium	5 (1.6%)	5 (2.8%)	1 (1.4%)	4 (1.9%)	22 (6.2%)	0.005
Vitamin A	4 (1.3%)	2 (1.1%)	0 (0.0%)	3 (1.4%)	3 (0.8%)	0.863
Folate	3 (1.0%)	0 (0.0%)	2 (2.8%)	4 (1.9%)	8 (2.3%)	0.226
Vit B12 tablets	18 (5.8%)	19 (10.8%)	15 (21.1%)	46 (21.3%)	124 (35.1%)	<0.001
Vit B12 spray	1 (0.3%)	3 (1.7%)	1 (1.4%)	3 (1.4%)	14 (4.0%)	0.015
Vit B12 injections	0 (0.0%)	0 (0.0%)	1 (1.4%)	5 (2.3%)	3 (0.8%)	0.033
Vitamin C	23 (7.4%)	23 (13.1%)	5 (7.0%)	28 (13.0%)	22 (6.2%)	0.015
Vitamin D	85 (27.4%)	49 (27.8%)	24 (33.8%)	67 (31.0%)	122 (34.6%)	0.284
Vitamin E	6 (1.9%)	6 (3.4%)	2 (2.8%)	4 (1.9%)	5 (1.4%)	0.626
Superfoods	6 (1.9%)	13 (7.4%)	2 (2.8%)	4 (1.9%)	20 (5.7%)	0.007
Other supplements	47 (15.2%)	21 (11.9%)	10 (14.1%)	26 (12.0%)	45 (12.7%)	0.800

Table S6. Mean daily dietary nutrient intakes: results from sensitivity analysis (includes vitamins, minerals, and supplements).

	Omnivores	Flexitarians	Pescatarians	Vegetarians	Vegans
Energy (kJ)	9511	9388	9277	9047	9132
Energy:BMR ratio	1.59	1.63	1.60	1.55	1.59
Energy:BMR ratio <1.2	0.18	0.17	0.18	0.21	0.20
Carbohydrate (%E)	37.8	44.8	46.4	48.9	49.2
Total sugars (%E)	17.4	19.9	20.1	20.6	20.2
Free sugars (%E)	7.67	7.85	8.02	8.99	7.40
Starch (%E)	20.5	25.0	26.4	28.4	30.8
Protein (%E)	17.8	15.1	14.2	12.6	12.2
Protein (g) per kg body weight	1.49	1.34	1.24	1.06	1.06
Fat (%E)	40.4	36.2	35.3	34.8	35.1
SFA (%E)	14.2	10.9	9.9	10.2	7.9
MUFA (%E)	16.0	14.9	14.7	14.1	14.9
PUFA (%E)	6.47	7.05	7.38	7.37	9.23
PS ratio	0.50	0.68	0.78	0.76	1.21
Cholesterol (mg)	415	248	217	168	20
N-3 fatty acids (g)	2.83	2.80	2.86	2.35	2.83
N-6 fatty acids (g)	13.8	15.1	15.7	15.9	20.2
Trans fatty acids (g)	1.45	0.95	0.77	0.80	0.38
Alcohol (%E)	2.85	2.85	3.16	2.72	2.23
Alcohol (g)	9.38	9.24	10.26	8.45	6.98
AOAC Fibre (g)	28.4	36.7	39.3	38.8	46.7
β-Carotene (μg)	3668	4240	4204	4039	4859
Retinol (μg)	997	466	272	259	102
Vitamin A (retinol equivalents) (μg)	1682	1261	1057	1009	989
Vitamin D (μg)	5.57	4.82	4.82	3.07	2.60
Thiamin (mg)	2.07	2.19	2.23	2.28	2.62
Riboflavin (mg)	2.26	2.03	1.96	1.94	1.80
Niacin equivalent (mg)	47.0	40.2	36.9	31.9	33.3
Vitamin C (mg)	151	170	175	167	193
Vitamin E (mg)	14.1	15.7	16.7	16.0	18.7
Vitamin B6 (mg)	2.30	2.15	2.05	1.90	2.04
Vitamin B12 (μg)	9.89	6.60	5.68	3.51	2.41
Folate (μg)	362	401	413	411	453
Pantothenic acid (mg)	8.25	7.42	6.90	6.42	5.84
Biotin (μg)	56.2	59.4	61.4	59.2	67.5
Sodium (mg)	1973	1816	1769	1761	1712
Potassium (mg)	3923	4072	4085	3836	4144
Calcium (mg)	976	1031	1055	1055	1022
Magnesium (mg)	363	414	430	413	478
Phosphorus (mg)	1619	1557	1537	1420	1397
Iron (mg)	13.9	15.1	15.4	15.2	17.6
Haem iron (mg)	1.05	0.49	0.30	0.16	0.18
Non-haem iron (mg)	12.8	14.5	15.1	15.1	17.3
Copper (mg)	1.80	1.86	1.86	1.81	2.20
Zinc (mg)	12.4	10.8	10.3	10.1	10.0
Chloride (mg)	3472	3269	3177	3166	3363

Iodine (μg)	232	203	200	135	89
Manganese (mg)	4.23	5.64	6.12	6.14	7.37
Selenium (μg)	79.7	67.5	66.4	43.2	41.3
